# Potential Roles of Cellular Senescence and Inflammaging in Prostate Cancer: Aging Microenvironment, Immune Remodeling, and Therapeutic Implications

**DOI:** 10.3390/cells15161457

**Published:** 2026-08-13

**Authors:** Qiuyang Zhang, Keyi Shen, Sen Liu

**Affiliations:** Department of Structural and Cellular Biology, Tulane University School of Medicine, New Orleans, LA 70112, USA; kshen1@tulane.edu (K.S.); sliu1@tulane.edu (S.L.)

**Keywords:** prostate cancer, aging, inflammaging, cellular senescence, senescence-associated secretory phenotype, tumor microenvironment, immune aging, Th17/Treg balance, IL-17/IL-23 axis, metabolic inflammation

## Abstract

**Highlights:**

**What are the main findings?**

- Aging reshapes the prostate tumor microenvironment through cellular senescence, chronic inflammation, immune remodeling, stromal dysfunction, vascular aging, and metabolic stress.
- Senescence-associated secretory programs and immune-aging networks create an inflammaging niche that may influence prostate cancer progression, immune evasion, and therapy response.

**What are the implications of the main findings?**

- Inflammaging should be considered an active biological context for prostate cancer progression, rather than merely a consequence of chronological aging.
- Age-appropriate models, biological-aging biomarkers, spatial profiling, and multi-omics approaches are needed to define and therapeutically target inflammaging-associated prostate cancer.

**Abstract:**

Prostate cancer is common in older men, yet mechanisms linking aging to tumor progression remain incompletely defined. Beyond genetic alterations, aging reshapes the prostate microenvironment through cellular senescence, chronic low-grade inflammation, immune dysfunction, stromal remodeling, metabolic stress, and impaired tissue repair, collectively promoting inflammaging. This persistent inflammatory state may create a permissive niche for tumor initiation, progression, immune evasion, and treatment resistance. Senescent epithelial and stromal cells release cytokines, chemokines, growth factors, matrix-remodeling enzymes, and extracellular vesicles through the senescence-associated secretory phenotype (SASP). In parallel, immune aging alters T-cell subsets, myeloid cells, macrophages, and anti-tumor surveillance. This review summarizes how SASP programs, Th17/Treg imbalance, IL-17/IL-23 signaling, myeloid remodeling, stromal aging, metabolic stress, and immune–stromal–epithelial crosstalk shape prostate cancer biology. We further discuss therapeutic implications, including cytokine modulation, senescence-directed therapy, metabolic intervention, and biomarker-guided strategies. This review highlights key knowledge gaps and proposes a framework for age-aware prostate cancer research, biomarker development, and therapeutic strategies.

## 1. Introduction: Aging as an Active Biological Context for Prostate Cancer

Prostate cancer is one of the most common malignancies in men and remains strongly associated with aging. In the United States, it is the most commonly diagnosed non-cutaneous malignancy among men, with an estimated 333,830 new cases and 37,400 deaths projected in 2026 [1,2,3]. Established risk factors include advanced age, family history, inherited genetic susceptibility, and African ancestry, whereas obesity, metabolic dysfunction, chronic inflammation, prostatitis/bacterial infection, and microbiota-related changes have been proposed as additional contributors or modifiers of disease risk and progression [2,4,5]. Although prostate cancer has traditionally been studied through tumor-intrinsic genetic and epigenetic alterations, aging also changes the biological context in which prostate cancer develops. The aged prostate is characterized by altered epithelial homeostasis, stromal remodeling, chronic inflammatory signaling, immune dysfunction, metabolic stress, vascular changes, and impaired tissue repair, all of which may influence tumor initiation, progression, immune escape, therapeutic response, and aggressive disease [1,2,4,6,7,8].

A key feature of biological aging is chronic, low-grade inflammation, commonly referred to as inflammaging. Inflammaging is driven by multiple cellular and systemic stressors, including cellular senescence, mitochondrial dysfunction, oxidative stress, endogenous danger signals, altered immune-cell composition, metabolic imbalance, and impaired resolution of inflammation [9,10,11,12]. Unlike acute inflammation, which is typically transient and protective, inflammaging is persistent and can gradually remodel tissue structure and function. In the prostate, this chronic inflammatory state may create a permissive microenvironment for tumor development and progression by altering the behavior of epithelial cells, stromal fibroblasts, endothelial cells, immune cells, and extracellular matrix (ECM) components.

Cellular senescence is one of the major biological processes linking aging to chronic tissue inflammation [13,14,15]. Senescent cells undergo stable growth arrest in response to stressors such as telomere dysfunction, DNA damage, oncogene activation, oxidative stress, and therapy-induced injury [14,16,17,18,19]. Although senescence can prevent proliferation of damaged cells and function as an important tumor-suppressive mechanism, persistent senescent cells remain metabolically active and can secrete cytokines, chemokines, growth factors, proteases, ECM-modifying enzymes, and extracellular vesicles through the senescence-associated secretory phenotype (SASP) [16,20,21,22,23,24]. Thus, with aging, senescence may shift from a protective response toward a chronic inflammatory and tumor-supportive process.

Immune aging further contributes to the inflammaging-associated prostate tumor microenvironment. Aging is associated with reduced naïve T-cell diversity, accumulation of memory and exhausted T-cell populations, altered regulatory T-cell activity, myeloid skewing, impaired antigen presentation, and increased production of inflammatory cytokines [25,26,27,28,29]. These immune changes may weaken anti-tumor surveillance while promoting chronic inflammation, immune suppression, angiogenesis, stromal remodeling, and resistance to therapy [30,31,32,33,34,35,36,37,38,39]. Among adaptive immune pathways, Th17/Treg imbalance and IL-17/IL-23-related signaling are particularly relevant because they can regulate epithelial, stromal, endothelial, and immune-cell responses through cytokine networks; however, their biological effects are context-dependent and may vary according to tumor stage, immune composition, tissue environment, and treatment status [40,41,42,43,44,45,46,47,48,49,50,51,52,53,54,55].

The IL-17/IL-23 axis represents one important inflammatory pathway that may connect immune aging to prostate cancer biology. IL-17 family cytokines can activate NF-κB, STAT3, chemokine expression, and tissue-remodeling programs in epithelial, stromal, endothelial, and immune cells. IL-23, produced primarily by myeloid and antigen-presenting cells, supports the maintenance of Th17-associated inflammatory responses. Together, IL-17- and IL-23-related signaling can sustain chronic inflammatory loops within tissues. In prostate cancer, these pathways may contribute to tumor-promoting inflammation, altered immune-cell recruitment, stromal remodeling, and disease progression. However, the biological effects of Th17-associated inflammation are context-dependent and may vary according to tumor stage, tissue environment, immune composition, and treatment status [40,41,42,43,44,45,46,47,55].

Stromal aging, vascular dysfunction, and metabolic stress are additional components of the aged prostate microenvironment. Aging fibroblasts and senescent stromal cells can generate inflammatory and matrix-remodeling signals, while endothelial aging may affect hypoxia, nutrient delivery, immune-cell trafficking, and angiogenesis [30,31,56,57,58,59,60,61,62,63,64,65,66,67]. Metabolic changes, including mitochondrial dysfunction, oxidative stress, obesity-associated inflammation, insulin resistance, and lipid dysregulation, can further amplify inflammatory signaling [68,69,70,71,72,73,74,75,76]. Together, these stromal, vascular, immune, and metabolic alterations may cooperate to create a tissue environment that supports prostate cancer progression and therapeutic resistance.

Importantly, aging-associated inflammation does not act through a single cell type or pathway. Instead, it reflects multicellular crosstalk among epithelial cells, immune cells, fibroblasts, endothelial cells, adipose-associated signals, ECM components, and systemic metabolic factors. This complexity has important implications for prostate cancer research because many preclinical studies rely on young animal models that may not accurately represent the aged tissue and immune environments in which human prostate cancer most commonly arises. Age-defined and temporally controlled prostate cancer models, including controlled Pten-knockout systems, provide more relevant approaches for studying how host age affects tumor initiation and progression [77,78,79]. Similarly, clinical studies that treat age only as a demographic variable may overlook biological differences among patients with distinct inflammatory, immune, senescent, stromal, and metabolic aging states.

Recent single-cell and spatial transcriptomic technologies provide new opportunities to define these aging-associated microenvironmental states [80,81]. Rather than treating immune cells, fibroblasts, endothelial cells, and epithelial cells as uniform compartments, these approaches reveal heterogeneous cell states, spatially organized inflammatory niches, and potential ligand–receptor interactions among tumor, stromal, vascular, and immune populations. Integrating these approaches with age-stratified models and human prostate cancer specimens may clarify how senescence, immune aging, stromal remodeling, and metabolic stress cooperate in situ.

**Figure 1:**
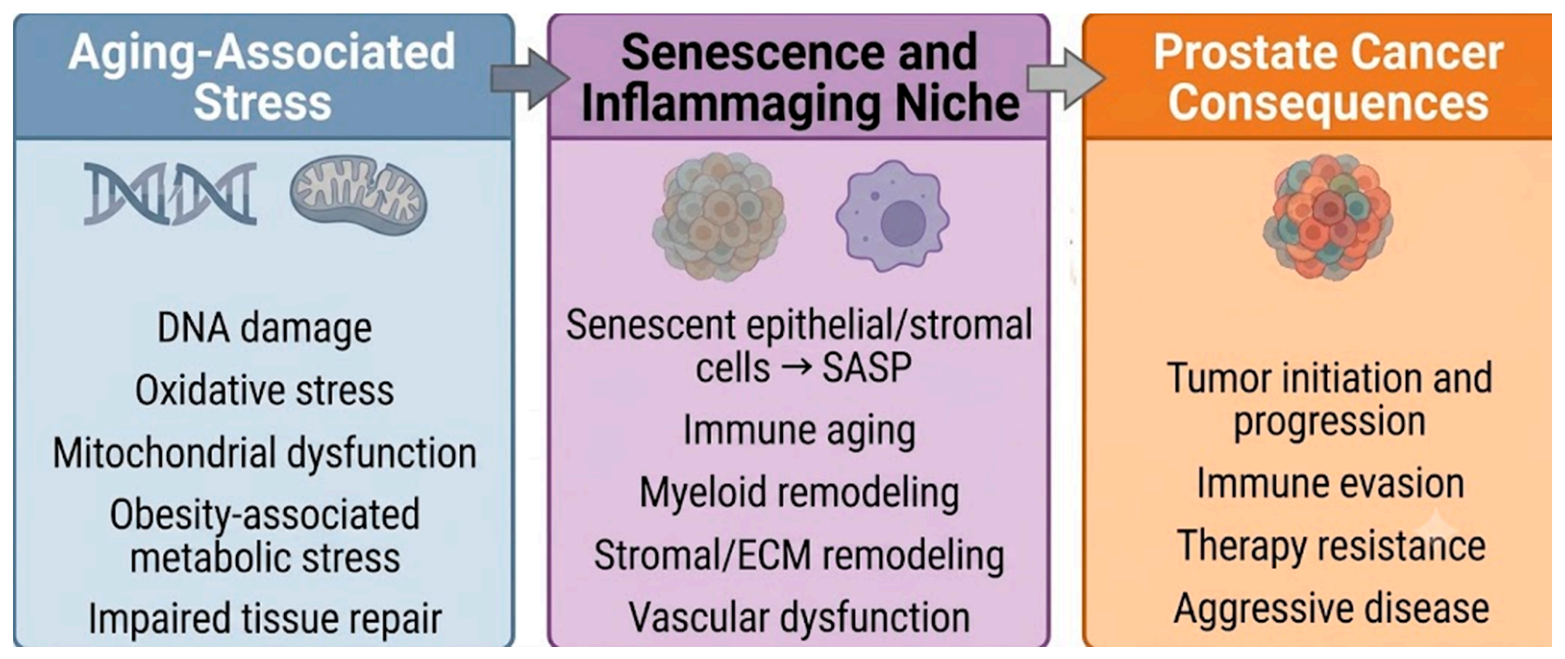
Aging-associated senescence and inflammaging in the prostate tumor microenvironment. Aging-associated stressors, including DNA damage, oxidative stress, mitochondrial dysfunction, obesity-associated metabolic stress, and impaired tissue repair, promote cellular senescence and chronic inflammatory remodeling. Senescent epithelial and stromal cells, together with immune aging, myeloid remodeling, stromal/ECM remodeling, and vascular dysfunction, contribute to an inflammaging niche. These interconnected processes may support prostate tumor initiation and progression, immune evasion, therapy resistance, and aggressive disease. Colored panels indicate the three conceptual stages, and arrows indicate proposed directional relationships among aging-associated stress, the senescence/inflammaging niche, and prostate cancer consequences. Selected key references: [10,14,15,21,25,26,27,28,29,30,68,75].

This review summarizes current knowledge of cellular senescence and inflammaging in prostate cancer, with a focus on the aging tumor microenvironment, immune remodeling, stromal dysfunction, metabolic stress, and therapeutic implications. We discuss SASP programs, immune aging, Th17/Treg imbalance, IL-17/IL-23-related inflammation, myeloid remodeling, ECM remodeling, and metabolic inflammatory signaling, while also highlighting unresolved questions and controversies. By focusing on aging as an active biological process rather than a passive clinical variable, this review aims to provide a framework for understanding how the aged prostate microenvironment contributes to prostate cancer progression and how this knowledge may inform biomarker development and future therapeutic strategies [4,6,8,9,29,30] (Figure 1).

## 2. Cellular Senescence in the Aging Prostate Microenvironment

### 2.1. Biological Features of Cellular Senescence

Cellular senescence is a stress-associated cell state characterized by stable proliferative arrest, altered metabolism, resistance to apoptosis, chromatin remodeling, and acquisition of a complex secretory phenotype. Senescence can be triggered by multiple intrinsic and extrinsic stressors, including telomere shortening, DNA damage, oncogene activation, oxidative stress, mitochondrial dysfunction, epigenetic disruption, inflammatory signaling, and therapeutic injury [14,15,17,18,19]. In young or acutely injured tissues, senescence can serve beneficial functions by preventing the expansion of damaged cells, limiting malignant transformation, promoting wound healing, and facilitating immune-mediated clearance of abnormal cells. However, with aging, senescent cells may accumulate because of persistent tissue stress and declining immune clearance. This accumulation can contribute to chronic inflammation, altered tissue architecture, and impaired regenerative capacity.

Senescent cells are commonly associated with activation of the p53–p21^CIP1^ and p16^INK4a^–RB tumor suppressor pathways, which enforce cell-cycle arrest through inhibition of cyclin-dependent kinase activity and suppression of E2F-dependent transcription [14,16,19,22,82,83]. These pathways are not mutually exclusive and may be activated to different degrees depending on the inducing stress, cell type, and tissue context. In addition to growth arrest, senescent cells often display persistent DNA damage response signaling, increased lysosomal activity, senescence-associated β-galactosidase activity, altered mitochondrial function, increased reactive oxygen species (ROS), changes in nuclear morphology, and formation of senescence-associated heterochromatin foci. Because no single marker universally defines senescence across all tissues and disease states, rigorous identification typically requires multiple complementary markers and functional evidence.

In the prostate, cellular senescence is particularly relevant because the gland undergoes lifelong hormonal regulation, repeated inflammatory exposure, age-associated stromal remodeling, and progressive changes in epithelial homeostasis [84,85,86,87]. Prostate epithelial cells, fibroblasts, endothelial cells, and immune cells may all acquire senescence-like features under chronic stress. These senescent or senescence-associated cell states may influence prostate tissue structure and tumor behavior through both cell-autonomous and non-cell-autonomous mechanisms. For example, senescence within epithelial cells may initially suppress malignant expansion by blocking proliferation of damaged cells, whereas senescence in surrounding stromal or immune compartments may alter epithelial behavior through paracrine inflammatory and growth-regulatory signals.

A key challenge in studying senescence in prostate cancer is distinguishing protective senescence from persistent, maladaptive senescence. Oncogene-induced senescence can prevent early tumor development by arresting premalignant cells. Therapy-induced senescence may also limit tumor-cell proliferation after DNA-damaging treatment or androgen pathway inhibition. However, when senescent cells are not efficiently cleared, their prolonged survival and secretory activity may create a tissue environment that supports tumor progression. Thus, senescence should not be viewed as a single fixed process, but as a dynamic biological state whose consequences depend on timing, cell type, immune context, and tissue microenvironment [18,20,88,89,90,91,92,93,94].

### 2.2. Senescence-Associated Secretory Phenotype

One of the most important non-cell-autonomous features of senescent cells is the SASP. The SASP consists of a heterogeneous mixture of soluble and insoluble mediators, including inflammatory cytokines, chemokines, growth factors, proteases, ECM-modifying enzymes, lipid mediators, and extracellular vesicles [16,20,21,22,23]. Rather than representing a fixed secretory program, SASP composition varies according to cell type, senescence trigger, duration of senescence, tissue environment, immune context, and therapeutic exposure.

Functionally, SASP mediators can be grouped into several major classes. Inflammatory cytokines such as IL-6, IL-1 family members, TNF-α, and TGF-β can activate NF-κB, STAT3, SMAD, and other stress-response pathways. Chemokines such as CCL2, CXCL1, CXCL2, CXCL8/IL-8, and CXCL12 can recruit monocytes, macrophages, neutrophils, lymphocytes, and other immune populations. Growth and angiogenic factors, including VEGF and GM-CSF, can support stromal activation, vascular remodeling, and tissue adaptation. Proteases and ECM-modifying factors, including MMPs, cathepsins, and matrix-remodeling enzymes, can alter tissue architecture and invasion. Extracellular vesicles and non-coding RNAs may provide an additional layer of intercellular communication, although their direct role in aging-specific prostate cancer inflammaging remains incompletely defined [15,16,20,21,22,23,24].

The SASP can have both beneficial and harmful consequences. In acute settings, SASP factors may reinforce senescence, recruit immune cells, promote clearance of damaged cells, and support tissue repair. However, during aging, persistent SASP activity may sustain chronic inflammation, impair immune surveillance, activate fibroblasts, remodel ECM, alter angiogenesis, and promote epithelial stress responses [15,16,20,21,22,23,24]. In the prostate, these effects are especially relevant because epithelial cells are embedded within a hormonally responsive and stromally active microenvironment. Thus, persistent SASP activity may provide a mechanism through which senescent epithelial or stromal cells influence neighboring non-senescent tumor and microenvironment cells.

Several pathways regulate SASP induction and maintenance. DNA damage response signaling, NF-κB activation, C/EBPβ, p38 MAPK, mTOR, cGAS-STING signaling, inflammasome activation, mitochondrial dysfunction, and ROS can all contribute to SASP regulation [16,22,82,83,95]. NF-κB and C/EBPβ are major transcriptional regulators of inflammatory SASP components, whereas mitochondrial dysfunction and cytosolic DNA sensing can amplify SASP activity through innate immune signaling. These pathways provide a mechanistic link among aging-associated cellular stress, senescence, and chronic inflammatory remodeling.

In prostate cancer, SASP factors may influence tumor biology by promoting inflammatory adaptation, immune-cell recruitment, stromal activation, ECM remodeling, angiogenesis, and treatment resistance [84,85,92,93,94,96,97]. IL-6 and related cytokines can activate STAT3 signaling; chemokines such as CCL2 and CXCL family members can recruit myeloid and lymphoid populations; MMPs and other proteases can remodel ECM; and growth or angiogenic mediators can support stromal and vascular changes. These mechanisms are not unique to prostate cancer, but they are highly relevant to aging-associated prostate tumor progression because they connect senescent-cell accumulation with multicellular microenvironmental remodeling.

Therapeutically, the SASP is attractive but challenging to target. Cancer therapies that induce DNA damage, oxidative stress, androgen deprivation, or cellular stress may generate senescent tumor or stromal cells. While therapy-induced senescence can suppress proliferation, persistent senescent cells may contribute to residual inflammatory and adaptive survival signals. This dual effect has led to interest in senescence-directed strategies, including senolytics that eliminate senescent cells and senomorphics that suppress harmful SASP programs without necessarily killing senescent cells [84,85,96,97,98,99,100,101,102,103,104]. For prostate cancer, these approaches remain investigational and will require careful definition of timing, cell type, disease stage, and patient selection.

A major unresolved issue is that SASP biology is highly context-dependent. Findings from fibroblast, epithelial, immune-cell, or therapy-induced senescence models may not be directly interchangeable, and much of the mechanistic evidence for SASP-driven tumor promotion remains preclinical. In human prostate cancer, SASP-related evidence is often correlative and based on marker expression, inflammatory signatures, or spatial association. Future studies should therefore define prostate cancer-specific SASP signatures by cell type, disease stage, treatment exposure, and host age, and determine which SASP components are protective, harmful, or therapeutically actionable.

### 2.3. Context-Dependent Senescence in Prostate Epithelial and Stromal Compartments

The relationship between senescence and prostate cancer is highly context-dependent. As a tumor-suppressive mechanism, senescence can prevent damaged or oncogene-stressed epithelial cells from continuing to proliferate, thereby limiting malignant transformation [18,88,89,92,93]. In prostate tissue, this protective response may occur after DNA damage, oxidative injury, oncogenic signaling, androgen-related stress, inflammation, or therapy-induced cellular injury. Activation of p53, p21^CIP1^, p16^INK4a^, RB, and related tumor-suppressor pathways can enforce growth arrest and maintain tissue integrity [86,87,92,93,94,105]. Thus, acute senescence followed by immune-mediated clearance may function as an important barrier to tumor initiation. However, senescence can also promote tumor progression when senescent cells persist and accumulate in aging tissue. In aged tissues, senescent epithelial, stromal, endothelial, or immune cells may remain metabolically active and sustain SASP-driven inflammatory and tissue-remodeling signals. Persistent SASP activity can influence neighboring non-senescent epithelial or tumor cells, recruit immune cells, activate fibroblasts, remodel ECM, promote angiogenesis, and alter tissue repair [14,15,20,21,23,84,85,96,97]. Therefore, senescence should not be viewed as uniformly protective or harmful. Its biological effect depends on timing, duration, cell type, immune clearance, tumor genotype, disease stage, and local microenvironment.

This duality is especially relevant in the prostate, where epithelial and stromal compartments are closely integrated. Prostate epithelial cells, including basal and luminal populations, are regulated by stromal fibroblasts, smooth muscle cells, endothelial cells, immune cells, ECM, nerves, and systemic hormonal and metabolic inputs [56,57,58,106,107,108]. Senescence-associated epithelial cells may reinforce local inflammatory signaling and alter stromal or immune-cell behavior, whereas senescent or activated stromal cells may produce cytokines, chemokines, growth factors, and matrix-remodeling enzymes that affect epithelial proliferation, survival, differentiation, and invasion. Thus, aging-associated senescence in prostate cancer is best understood as a tissue-level process rather than a feature of one cell type alone.

Stromal senescence may be particularly important because prostate fibroblasts and smooth muscle cells help maintain epithelial organization, androgen responsiveness, ECM structure, and paracrine growth control [30,31,32,56,57,58,106,107,108,109,110,111,112,113]. With aging, stromal fibroblasts may acquire senescence-associated or activated phenotypes characterized by altered secretory activity, inflammatory signaling, ECM remodeling, and reduced capacity to maintain normal epithelial homeostasis. These stromal changes may overlap with, but are not identical to, cancer-associated fibroblast biology. Senescent fibroblasts are typically growth arrested and defined by stress-associated cell-cycle arrest and SASP production, whereas cancer-associated fibroblasts are heterogeneous activated stromal cells that may or may not be senescent. In aged prostate tissue, these populations may coexist and cooperate to promote chronic inflammation, matrix remodeling, tissue stiffness, immune modulation, and tumor-supportive stromal niches [30,31,32,107,108,109,110,111,112,113].

Endothelial and immune-cell senescence add additional layers of complexity. Senescent endothelial cells may contribute to vascular dysfunction, altered permeability, impaired angiogenic balance, hypoxia, and modified immune-cell trafficking [62,63,64,65,66,67]. Because tumor progression depends on oxygen availability, nutrient delivery, immune-cell access, and vascular remodeling, endothelial aging may influence both tumor growth and therapeutic response. Aging immune cells may also show senescence-like or exhaustion-like features, including impaired effector function, altered antigen presentation, myeloid skewing, and persistent inflammatory mediator production [25,26,27,28,29,114,115,116,117]. This combination of reduced immune surveillance and sustained inflammatory signaling is central to inflammaging-associated prostate cancer biology.

Prostate cancer provides disease-specific examples of this senescence duality. In PTEN-deficient prostate tumorigenesis, senescence-associated programs can initially restrain tumor progression through p53-, p21-, p16^INK4a^-, RB-, and p27-related pathways [86,87,92,93,105]. However, these protective programs can be bypassed or reprogrammed during progression. In prostate cancer models, modulation of senescence-regulatory pathways and tumor-associated myeloid cells can alter senescence outcomes and support disease progression [86,92,93,94,105]. These findings support the broader concept that senescence in prostate cancer is not simply tumor suppressive or tumor promoting, but depends on tumor genotype, immune context, stromal state, and disease stage.

The therapeutic implications are substantial. Complete suppression of senescence may be undesirable because senescence can restrict malignant transformation and contribute to treatment response. Conversely, persistent senescent cells and chronic SASP activity may promote inflammatory remodeling, immune dysfunction, invasion, and therapy resistance. Therefore, senescence-directed strategies in prostate cancer should aim to distinguish beneficial acute senescence from maladaptive persistent senescence. Key unresolved questions include which senescent cell populations accumulate during aging, which SASP components are most relevant to prostate cancer progression, whether senescent cells are efficiently cleared after therapy, and which patients exhibit senescence-associated inflammatory signatures that can be safely targeted [98,99,100,101,102,103,104]. The major senescence-associated cell types and potential effects in the aging prostate microenvironment are summarized in Table 1.

## 3. Immune Aging and Inflammaging in Prostate Cancer

### 3.1. Immunosenescence and Impaired Immune Surveillance

The immune system undergoes substantial remodeling during aging, a process commonly referred to as immunosenescence. This process affects both innate and adaptive immune compartments and is characterized by reduced immune diversity, altered immune-cell function, chronic inflammatory activation, and impaired immune resolution [25,26,27,28]. In the adaptive immune system, aging is associated with thymic involution, reduced generation of naïve T cells, contraction of T-cell receptor diversity, expansion of memory and terminally differentiated T-cell populations, and increased features of T-cell dysfunction or exhaustion [33,34,35]. These changes may limit the ability of the aged immune system to recognize and eliminate emerging malignant cells.

Effective anti-tumor immunity requires coordinated antigen presentation, T-cell priming, cytotoxic effector function, immune-cell trafficking, and immune memory [25,26,27,28,33,34,35]. Aging can disrupt each of these steps. Dendritic cells and other antigen-presenting cells may show altered antigen uptake, processing, migration, and co-stimulatory function. CD8^+^ T cells may exhibit reduced proliferative capacity, impaired cytokine production, diminished cytotoxic activity, and increased expression of inhibitory receptors. CD4^+^ T-cell subsets may shift in frequency and function, altering the balance between anti-tumor immunity and tumor-promoting inflammation. Together, these age-associated changes may reduce immune surveillance and allow prostate epithelial lesions or established tumor cells to persist under reduced immune pressure.

At the same time, immunosenescence does not simply represent immune deficiency. Aging is also associated with increased basal inflammatory tone, sometimes described as sterile chronic inflammation or inflammaging. Therefore, the aged immune system can be simultaneously less effective at tumor elimination and more prone to chronic inflammatory activation [9,10,11,12,26,27,28,29,36,117]. This paradox is highly relevant to prostate cancer, where chronic immune-cell infiltration and inflammatory cytokine production may coexist with weak anti-tumor cytotoxicity. In this setting, immune aging may support tumor progression not only by reducing protective surveillance but also by generating inflammatory signals that remodel the tumor microenvironment.

In prostate cancer, the immune microenvironment is typically complex and heterogeneous. Tumors may contain T cells, macrophages, myeloid-derived suppressor cells, dendritic cells, neutrophils, natural killer cells, mast cells, and B cells, with variable functional states depending on tumor stage, tissue location, treatment history, and host factors [30,31,32,33,34,35,37,38,39]. In older patients, these immune populations are further shaped by systemic aging, metabolic status, comorbidities, prior inflammation, and therapy exposure. Thus, understanding prostate cancer immunity requires considering not only tumor-intrinsic immune escape mechanisms but also the aged host immune environment in which the tumor develops.

### 3.2. Chronic Inflammation and Cytokine Imbalance

Inflammaging is characterized by persistent, low-grade inflammatory signaling that occurs in the absence of acute infection. This chronic inflammatory state is driven by multiple aging-associated processes, including cellular senescence, mitochondrial dysfunction, oxidative stress, endogenous damage-associated molecular patterns, altered tissue repair, metabolic stress, and impaired clearance of damaged cells [9,10,12,26,28]. In the prostate microenvironment, chronic inflammation may influence epithelial behavior, stromal activation, immune-cell recruitment, angiogenesis, ECM remodeling, and therapeutic response.

Several cytokine and chemokine pathways have been implicated in aging-associated inflammation and prostate cancer progression. IL-6 is one of the best-studied inflammatory cytokines in cancer biology and can activate JAK/STAT3 signaling, which has been linked to tumor-cell survival, proliferation, inflammatory adaptation, angiogenesis, and treatment resistance [20,21,22,95]. TNF-α can activate NF-κB and other stress-response pathways, contributing to inflammatory gene expression, immune-cell recruitment, and stromal remodeling. IL-1 family cytokines can amplify innate immune activation and inflammatory tissue injury. TGF-β has context-dependent roles, functioning in some settings as a suppressor of epithelial proliferation and in others as a driver of immune suppression, fibroblast activation, ECM remodeling, and invasive behavior [30,31,33,34,35,37,38,39].

Chemokines are also central mediators of the inflammatory tumor microenvironment. CCL2, CXCL1, CXCL2, CXCL8/IL-8, CXCL12, and related chemokine pathways can regulate recruitment of monocytes, macrophages, neutrophils, lymphocytes, and other immune-cell populations [30,31,32]. In aged tissues, persistent chemokine production may reinforce immune-cell infiltration and chronic inflammation. In prostate cancer, chemokine signaling may also influence stromal remodeling, angiogenesis, tumor-cell migration, and immune suppression. These pathways provide important links between senescence-associated secretory programs, immune-cell recruitment, and tumor-supportive inflammation.

IL-17- and IL-23-related inflammatory signaling represents one important example of cytokine imbalance in prostate cancer, but this pathway is discussed in detail in Section 3.3 in the context of Th17/Treg biology. In brief, IL-17 and IL-23 can contribute to chronic inflammatory circuits by influencing epithelial, stromal, endothelial, and immune-cell responses. Their effects are context-dependent and may vary according to tumor stage, immune composition, cytokine milieu, and local tissue environment [40,41,42,43,44,45,46,47,55].

Importantly, cytokine imbalance in the aged prostate microenvironment should not be viewed as the effect of one isolated mediator. Instead, inflammatory cytokines operate as interconnected networks. IL-6, IL-17, TNF-α, IL-1, IL-23, TGF-β, and chemokines may cooperate or counter-regulate one another depending on cellular context. These pathways can also intersect with androgen receptor signaling, metabolic stress, senescence-associated programs, and stromal activation [30,31,33,34,35,37,38,39,40,41,42,43,44,45,46,47,48,49,50,51,52,53,54,55]. Therefore, therapeutic targeting of inflammaging in prostate cancer will likely require careful patient stratification and a detailed understanding of dominant inflammatory circuits in each biological context.

### 3.3. Th17/Treg Balance and IL-17/IL-23 Signaling in Aging-Associated Prostate Cancer

CD4^+^ T-cell subsets are important regulators of tissue inflammation and tumor immunity. Among these subsets, Th17 cells and regulatory T cells are particularly relevant to chronic inflammatory diseases, aging-associated inflammation, and cancer. Th17 cells produce IL-17A, IL-17F, IL-21, IL-22, and other inflammatory mediators, whereas regulatory T cells suppress immune activation and maintain immune tolerance through mechanisms involving FOXP3, IL-10, TGF-β, CTLA-4, and related pathways [48,49,50,51,52,55]. The balance between Th17-associated inflammation and Treg-mediated immune suppression can shape both inflammatory tissue remodeling and anti-tumor immunity.

Aging may alter the differentiation, balance, and function of Th17 and Treg populations. Chronic antigenic stimulation, systemic inflammatory tone, altered antigen-presenting cell function, metabolic stress, microbial changes, and tissue damage can influence CD4^+^ T-cell polarization during aging [25,26,27,28,43,114,115,116,117]. In some contexts, aging-associated inflammation may favor Th17-related responses, whereas increased Treg activity or altered Treg function may suppress anti-tumor effector immunity. The resulting immune state may therefore combine chronic inflammatory signaling with reduced tumor immune surveillance. This paradox is central to inflammaging-associated prostate cancer, in which the aged immune system may be inflammatory but not effectively tumoricidal.

Prostate cancer-specific studies support a role for IL-17-related signaling in tumor development and progression. IL-17 signaling has been shown to promote formation and growth of prostate adenocarcinoma in mouse models, including PTEN-related prostate tumorigenesis [118]. IL-17 has also been reported to promote castration-resistant prostate cancer by creating an immunotolerant and pro-angiogenic tumor microenvironment [41]. Mechanistically, IL-17 can promote prostate cancer progression through MMP7-associated epithelial-to-mesenchymal transition, linking inflammatory cytokine signaling to matrix remodeling and invasive tumor behavior [119]. These studies suggest that IL-17 is not merely a general inflammatory marker but can directly regulate tumor and microenvironmental programs relevant to prostate cancer progression.

Age may further modify the biological consequences of IL-17 signaling. In age-defined murine studies, CD4^+^ helper 17 cell responses from aged mice promoted prostate cancer cell migration and invasion, supporting the concept that aged immune environments can shift Th17-associated inflammation toward more pathogenic tumor-microenvironment interactions [43]. This finding is important because many cancer immunology studies rely on young hosts, whereas human prostate cancer most commonly arises in older men. Thus, the impact of IL-17/Th17 signaling in prostate cancer should be interpreted in relation to host age, inflammatory burden, tissue context, and immune-cell composition.

IL-23-related signaling provides another mechanistic link between Th17 biology, myeloid cells, and prostate cancer progression. IL-23 is produced primarily by myeloid and antigen-presenting cells and supports maintenance of Th17-associated inflammatory responses. In genetically engineered prostate cancer models, BATF-dependent Th17 cells were shown to act through the IL-23R pathway to promote prostate adenocarcinoma initiation and progression, and anti-IL-23p19 treatment reduced IL-23R- and IL-17-positive cells in Pten-deficient mouse prostate stroma [40]. This study also provided human translational support by showing positive correlations between BATF and IL23A/IL23R mRNA levels in human prostate tumors. In a complementary study focused on castration-resistant disease, myeloid-derived suppressor cell-secreted IL-23 promoted androgen-independent prostate cancer growth through activation of androgen receptor signaling, with supporting evidence from mouse models, prostate cancer cell systems, and human CRPC specimens [44]. Together, these studies identify IL-23 as a prostate cancer-relevant inflammatory pathway linking myeloid remodeling, Th17-associated immunity, androgen signaling, and disease progression.

Human prostate tissue studies provide additional, although largely correlative, support for this pathway. Treg and Th17 infiltration has been described in benign prostatic hyperplasia and prostate cancer in association with inflammatory responses, and IL-17A/IL-17RA expression patterns have been linked to prostate cancer with lymph-node metastasis [46,47]. These observations suggest clinical relevance of IL-17/IL-23-related inflammation in prostate cancer, but they do not by themselves establish causality. Therefore, evidence from human specimens should be interpreted as supportive of pathway relevance, whereas genetically engineered and intervention-based models provide stronger mechanistic evidence.

Importantly, IL-17 and Th17 responses are not uniformly tumor promoting. Studies in other tumor types have reported that endogenous IL-17 can reduce tumor growth and metastasis, and that tumor-specific Th17 cells can promote cytotoxic CD8^+^ T-cell activation and protective anti-tumor immunity [53,54]. These findings indicate that IL-17 biology depends strongly on tumor type, host immune competence, cytokine context, disease stage, microbiota, tissue environment, and effector-cell recruitment. Thus, IL-17/Th17 signaling should not be treated as a universally harmful inflammatory pathway across all cancers.

In prostate cancer, however, the available evidence more strongly supports tumor-promoting roles for IL-17/IL-23-related inflammation, particularly in chronic inflammation, angiogenesis, immune remodeling, MMP7-associated invasion, castration resistance, and disease progression [40,41,42,43,44,45,46,47,55,118,119]. A balanced interpretation is that IL-17/IL-23 signaling may be pathogenic in prostate cancer when it occurs within aged, myeloid-rich, stromally remodeled, or immunosuppressive niches, but may not have the same consequences in other tumor types or immune contexts. This distinction is important for therapeutic translation because broad IL-17 or IL-23 blockade without biomarker selection could suppress protective immunity in some patients while benefiting only those with dominant IL-17/IL-23-driven inflammatory programs.

Key unresolved questions remain. It is not yet clear how aging modifies Th17/Treg balance in human prostate tumors, whether IL-17/IL-23-associated signatures predict progression or treatment response, or which patients would benefit from IL-17/IL-23-directed intervention. Future studies should integrate age-stratified human cohorts, spatial immune profiling, myeloid-cell analysis, cytokine measurements, and functional models to determine whether IL-17/IL-23 signaling marks a therapeutically actionable inflammaging phenotype in prostate cancer.

### 3.4. Myeloid Remodeling During Aging

Myeloid cells are major contributors to inflammaging and the tumor microenvironment. Aging is associated with myeloid-biased hematopoiesis, altered monocyte and macrophage function, impaired antigen presentation, increased basal inflammatory activation, and reduced resolution of tissue inflammation [25,26,27,28,36,114,115,116,117]. These changes can promote persistent cytokine production and inflammatory remodeling in multiple tissues. In cancer, age-associated myeloid remodeling may support immune suppression, angiogenesis, matrix remodeling, tumor-cell survival, and resistance to therapy.

Tumor-associated macrophages are among the most abundant immune populations in many solid tumors, including prostate cancer. These cells can display diverse functional states rather than a simple binary M1/M2 classification. Depending on microenvironmental cues, macrophages may produce inflammatory cytokines, growth factors, angiogenic mediators, proteases, immunosuppressive molecules, and chemokines [30,31,32]. In prostate cancer, macrophage-rich microenvironments have been associated with tumor progression, immune suppression, and therapy resistance in several studies. Aging may further influence macrophage function by altering metabolic state, phagocytic capacity, cytokine production, and responsiveness to tissue damage.

Myeloid-derived suppressor cells are another important population in tumor-associated immune suppression. These cells can inhibit T-cell activation and effector function through mechanisms involving arginine metabolism, ROS, nitric oxide, immunosuppressive cytokines, and checkpoint-related pathways [30,32,33,34,35,37,38,39,44]. In aged hosts, expansion of immature or suppressive myeloid populations may be favored by chronic inflammation, altered hematopoiesis, and tumor-derived factors. Their accumulation in prostate cancer may contribute to poor anti-tumor immunity and limited response to immunotherapy.

Dendritic cells may also be affected by aging and tumor-associated inflammation. Effective dendritic cell function is required for antigen presentation and T-cell priming. Aging can impair dendritic cell migration, antigen presentation, cytokine production, and co-stimulatory signaling [25,26,27,28,33,34,35,114,115,116,117]. Within the prostate tumor microenvironment, suppressive cytokines, metabolic stress, tumor-derived factors, and chronic inflammation may further weaken dendritic cell function. This may reduce productive anti-tumor T-cell priming and promote immune tolerance.

Neutrophils and inflammatory monocytes may also contribute to the aged prostate tumor microenvironment. These cells can produce cytokines, proteases, ROS, and extracellular traps and may influence angiogenesis, matrix remodeling, and tumor-cell invasion [25,26,27,28,30,31,114,115,116,117]. Although their roles in prostate cancer require further study, they represent important components of innate immune remodeling. Because aging is associated with altered innate immune responsiveness and persistent inflammatory activation, these myeloid populations may help sustain inflammaging-associated tumor-promoting niches.

Recent single-cell and spatial studies further show that prostate cancer-associated myeloid cells are heterogeneous and cannot be fully represented by broad macrophage or MDSC categories. Integrated single-cell and spatial analyses have identified multiple macrophage subpopulations in human prostate cancer, including inflammatory, antigen-presenting, and M2-like states, while studies of primary and metastatic prostate cancer have identified SPP1+/TREM2+ tumor-associated macrophages enriched in metastatic tumors [80,120]. These findings suggest that aging-related myeloid remodeling should be analyzed at the level of cell states, spatial location, and interaction with tumor and stromal niches rather than by macrophage abundance alone.

Overall, myeloid remodeling provides a major link between aging, inflammation, and prostate cancer progression. Myeloid cells can produce cytokines such as IL-6, IL-1β, TNF-α, IL-10, TGF-β, and IL-23; recruit additional immune cells through chemokines; suppress T-cell responses; remodel ECM; and support angiogenesis. These functions place myeloid cells at the center of aging-associated inflammatory crosstalk in the prostate tumor microenvironment [30,32,33,34,35,37,38,39,44].

### 3.5. Transcriptional Control of Inflammatory Immune States and NF-κB/STAT3 Signaling

Immune aging and inflammaging are regulated not only by changes in immune-cell abundance, but also by changes in transcriptional and epigenetic programs that determine immune-cell identity and function. T-cell differentiation, macrophage polarization, dendritic cell activation, and inflammatory cytokine production are controlled by coordinated transcription factor networks. In aging tissues, chronic inflammatory stimuli, metabolic stress, antigen exposure, and altered cytokine environments may reshape these transcriptional programs and produce persistent inflammatory or dysfunctional immune states [25,26,27,28,114,115,116,117].

In T cells, transcription factors such as T-bet, GATA3, RORγt, FOXP3, STAT3, STAT5, IRF4, and AP-1 family members regulate differentiation into effector, regulatory, and inflammatory subsets. Th17 differentiation requires coordinated cytokine signaling and transcriptional programming involving STAT3, RORγt, IRF4, BATF, and related factors [48,49,50,51,52,55]. Tregs depend on FOXP3 and associated regulatory networks. The balance between these programs is influenced by cytokines such as IL-6, TGF-β, IL-1β, IL-21, IL-23, IL-2, and inflammatory metabolic cues. Aging-associated changes in these signals may therefore alter T-helper cell balance and inflammatory potential.

BATF is an established transcriptional regulator involved in Th17 differentiation and inflammatory T-cell programming. In the context of this review, BATF can be discussed as an example of how transcriptional regulation shapes immune-cell inflammatory states. Published studies have shown that BATF-dependent Th17 programs and IL-23/IL-23R-related signaling can contribute to prostate cancer-associated inflammation and tumor progression [40,44,55]. However, the broader relationship among immune-cell transcriptional states, biological aging, metabolic stress, and prostate tumor behavior remains an active area of investigation. For this reason, this review does not present BATF as a single central driver of aging-associated prostate cancer, but rather as one component of a broader inflammatory transcriptional network.

In myeloid cells, transcriptional regulators such as NF-κB, STAT1, STAT3, STAT6, HIF-1α, C/EBPβ, IRF family members, and nuclear receptors can control inflammatory activation, immune suppression, antigen presentation, and metabolic adaptation. These programs are particularly relevant to inflammaging because aged and tumor-associated myeloid cells are exposed to cytokines, pattern-recognition receptor ligands, oxidative stress, senescence-associated mediators, metabolic cues, and tumor-derived factors [26,30,32]. Such signals can promote cytokine production, chemokine expression, myeloid recruitment, immune suppression, and stromal remodeling in the prostate tumor microenvironment.

Within this broader transcriptional network, NF-κB and STAT3 serve as central inflammatory convergence hubs. NF-κB integrates signals from inflammatory cytokines, pattern-recognition receptors, DNA damage, ROS, and senescence-associated mediators, whereas STAT3 is activated downstream of IL-6 family cytokines and other inflammatory pathways [26,30,32,82,83,93]. Together, these pathways can promote cytokine production, chemokine expression, myeloid recruitment, stromal remodeling, immune suppression, tumor-cell survival, and therapy resistance. Because NF-κB and STAT3 are activated by multiple upstream signals, they are best viewed as shared inflammatory convergence nodes rather than pathway-specific markers of any single cell type or mediator.

Epigenetic regulation also contributes to immune-cell aging. DNA methylation changes, histone modifications, chromatin accessibility, and non-coding RNAs can affect immune-cell differentiation, inflammatory memory, and exhaustion-like states [25,26,27,28,114,115,116,117]. Trained immunity in innate immune cells and chronic antigen stimulation in adaptive immune cells may establish durable immune states that persist even after the original stimulus has changed. In prostate cancer, such stable inflammatory or suppressive immune states may influence tumor progression and treatment response.

Understanding transcriptional control of immune aging has practical implications. Cell-type-specific immune signatures may help distinguish patients with inflammatory, immunosuppressive, senescent, or metabolically stressed tumor microenvironments. These signatures could support biomarker development and guide selection of therapies targeting cytokines, immune checkpoints, myeloid cells, senescence-associated inflammation, or metabolic pathways [33,34,35,37,38,39]. Future studies using single-cell transcriptomics, spatial profiling, epigenomic analysis, and age-stratified models will be important for defining how immune transcriptional states shape the aging prostate tumor microenvironment.

#### Section Summary

Immune aging links impaired surveillance with persistent inflammatory activation in prostate cancer. Changes in T-cell diversity, Th17/Treg balance, myeloid activity, antigen presentation, and cytokine signaling may promote chronic inflammation, immune suppression, stromal remodeling, and therapy resistance. These pathways operate as an integrated multicellular network rather than isolated drivers, and cell-type-specific studies will be essential for biomarkers and therapeutic development (Table 2).

## 4. Stromal Aging, Extracellular Matrix Remodeling, and Prostate Tumor Progression

### 4.1. Aging Fibroblasts and Cancer-Associated Fibroblasts

The prostate is a highly stromal organ in which epithelial cells are embedded within a complex microenvironment composed of fibroblasts, smooth muscle cells, endothelial cells, immune cells, nerves, ECM, and soluble paracrine factors. During aging, this stromal compartment undergoes progressive remodeling that can alter epithelial homeostasis and influence prostate cancer development. Stromal aging is characterized by changes in fibroblast function, ECM organization, inflammatory signaling, tissue stiffness, paracrine growth regulation, and response to injury [56,57,58,106,107,108]. These changes may create a permissive tissue environment in which premalignant or malignant epithelial cells acquire growth and survival advantages.

Fibroblasts are central regulators of prostate tissue architecture. In normal prostate tissue, stromal fibroblasts and smooth muscle cells help maintain epithelial differentiation, androgen responsiveness, basement membrane integrity, and paracrine growth control. With aging, fibroblasts may acquire senescence-associated or activated phenotypes marked by altered cytokine production, growth factor secretion, ECM remodeling, and reduced ability to maintain normal epithelial organization [56,57,58,106,107,108]. These changes can disrupt stromal–epithelial communication and contribute to chronic tissue inflammation.

Consistent with the SASP program described above, senescent stromal fibroblasts can generate inflammatory, growth-regulatory, and matrix-remodeling signals that influence epithelial proliferation, immune-cell recruitment, angiogenesis, and ECM remodeling [14,15,56,57,58,106,107,108]. As discussed above, persistent stromal senescence may shift from an acute stress response toward a chronic inflammatory state that supports tumor progression. Thus, stromal senescence represents an important mechanism through which aging may affect prostate cancer biology.

CAFs are another major component of the prostate tumor microenvironment. CAFs are functionally heterogeneous and can arise from local fibroblasts, smooth muscle cells, pericytes, mesenchymal progenitors, or other stromal sources depending on tissue context [30,31,107,108,109,113]. In prostate cancer, CAFs can generate paracrine, inflammatory, and matrix-remodeling signals that support tumor-cell survival, local invasion, angiogenesis, immune suppression, and therapeutic resistance. CAFs can also alter androgen receptor signaling and epithelial differentiation through paracrine mechanisms, although the precise effects depend on tumor stage and stromal subtype. Recent single-cell studies also support this heterogeneity by identifying distinct CAF-related states in prostate cancer, including peri-epithelial fibroblast programs and CAF signatures associated with aggressive cribriform/intraductal carcinoma and worse clinical outcomes. Single-cell and spatial approaches now further support this heterogeneity by distinguishing fibroblast, pericyte, and CAF-related states within prostate tumors, emphasizing that aging-associated stromal remodeling may involve specific fibroblast programs rather than uniform fibroblast activation [80].

Aging may influence CAF biology through several interconnected mechanisms [29,30,31,56,57,58]. Aged fibroblasts may be more prone to inflammatory activation or SASP-like programs, while chronic low-grade inflammation, altered tissue mechanics, and changes in matrix composition may reinforce CAF phenotypes. In addition, impaired immune clearance of senescent or damaged stromal cells may allow tumor-supportive stromal niches to persist. Together, these processes suggest that aging may not only affect epithelial tumor cells directly but also reshape the stromal context in which tumor cells evolve.

The distinction between senescent fibroblasts and CAFs is important but not absolute. Senescent fibroblasts are typically growth arrested and defined by stress-associated cell-cycle arrest and SASP production, whereas CAFs are activated stromal cells with diverse functional states that may or may not be senescent. Nevertheless, these populations can share overlapping features, including secretory activity, ECM remodeling, and paracrine support of epithelial cells. In aged prostate tissue, senescent stromal cells and CAF-like cells may coexist and cooperate to promote chronic inflammation and tumor-supportive remodeling [14,15,30,31,32,107,108,109,110,111,112,113].

Understanding stromal aging in prostate cancer is clinically relevant because the tumor stroma can influence tumor growth, invasion, immune-cell exclusion, drug delivery, and therapeutic response. Stromal signatures have been associated with prostate cancer aggressiveness in multiple studies, and stromal-rich or desmoplastic tumor regions may create physical and biochemical barriers to effective treatment [30,31,32,108,109,110,111,112,113]. Therefore, defining how aging alters stromal fibroblast states may provide new opportunities for biomarker development and therapeutic intervention.

### 4.2. Extracellular Matrix Remodeling

The ECM is a dynamic structural and signaling network that regulates tissue architecture, cell adhesion, growth factor availability, mechanical tension, migration, and cellular differentiation. In the prostate, the ECM provides essential support for epithelial organization and stromal–epithelial communication. Aging can alter ECM composition, crosslinking, stiffness, proteolytic remodeling, and spatial organization, thereby changing the biochemical and mechanical properties of the tissue microenvironment [59,60,61,121,122,123].

ECM remodeling is a hallmark of cancer progression. In prostate cancer, changes in collagen deposition, basement membrane integrity, fibronectin expression, laminin organization, hyaluronan accumulation, and matrix stiffness can influence tumor-cell behavior [56,59,60,121,122]. Increased matrix stiffness can activate mechanotransduction pathways involving integrins, focal adhesion kinase, SRC family kinases, YAP/TAZ, Rho GTPases, and cytoskeletal remodeling. These pathways can regulate cell survival, migration, invasion, and resistance to therapy [59,60,121,122,123]. Thus, the ECM is not merely a passive scaffold but an active regulator of tumor biology.

Aging can promote ECM remodeling through several mechanisms. Senescent fibroblasts and activated stromal cells can produce MMPs, cathepsins, lysyl oxidases, collagens, fibronectin, and other matrix-associated factors. Chronic inflammation can further stimulate matrix remodeling through cytokines such as IL-6, TNF-α, TGF-β, and IL-1 family members. Oxidative stress and glycation can alter matrix crosslinking and stiffness. Impaired tissue repair may lead to disorganized matrix deposition after injury or inflammation [29,60,61,86,123]. Together, these age-associated changes may modify the physical and signaling environment surrounding prostate epithelial and tumor cells.

MMPs are particularly important mediators of ECM remodeling. MMPs can degrade basement membrane and interstitial matrix components, release matrix-bound growth factors, and generate bioactive matrix fragments. In prostate cancer, MMP activity has been associated with invasion, angiogenesis, and metastatic potential in many studies [56,59,60,61,108,123]. In aging tissues, increased inflammatory signaling and senescent stromal activity may enhance MMP production, contributing to tissue remodeling and local invasion. However, MMPs also have context-dependent roles, and broad inhibition of MMP activity has historically been challenging in cancer therapy. A more precise understanding of cell-type-specific MMP regulation may be needed for future therapeutic approaches.

Prostate cancer-specific studies also support a direct link among senescence, matrix remodeling, and disease progression [124]. In PTEN-deficient prostate cancer models, TIMP1 has been identified as a molecular switch that restrains the tumor-promoting effects of senescence. Loss of TIMP1 reprograms senescence-associated secretory activity through MMP-related matrix remodeling, promotes metastatic behavior, and has been associated with docetaxel resistance and worse clinical outcomes in prostate cancer. These findings connect senescence biology directly to ECM remodeling and therapeutic resistance in a prostate cancer-specific context.

TGF-β signaling is another major regulator of stromal and ECM remodeling. In normal tissues and early tumorigenesis, TGF-β can suppress epithelial proliferation and maintain tissue homeostasis. In later-stage cancer or chronically inflamed tissue, TGF-β may promote fibroblast activation, immune suppression, ECM deposition, epithelial stress responses, and invasive behavior [30,107,108,110,111]. Aging-associated increases in TGF-β activity may therefore contribute to fibrotic remodeling and tumor-supportive stromal changes. In prostate cancer, TGF-β-related stromal signaling may influence tumor progression, immune exclusion, and response to therapy.

ECM remodeling also affects immune-cell trafficking and function. Dense or disorganized matrix can restrict T-cell infiltration, alter macrophage localization, and create physical barriers that limit drug delivery. Matrix components can also signal directly to immune cells through integrins and other receptors, influencing immune activation or suppression [30,59,60,61,111,123]. In the aged prostate tumor microenvironment, ECM remodeling may therefore cooperate with immune aging to create niches that support immune evasion. This is especially relevant because prostate cancer often exhibits limited response to immune checkpoint blockade in unselected patients, and stromal or matrix barriers may contribute to this resistance.

In addition, ECM remodeling can influence tumor-cell adaptation to therapeutic stress. Changes in matrix stiffness and integrin signaling can activate survival pathways that reduce sensitivity to androgen receptor-directed therapy, chemotherapy, radiation, or targeted agents. Matrix-rich tumor regions may also show altered vascularization and hypoxia, which can further affect therapeutic response [59,60,61,108,113,123]. Therefore, age-associated ECM remodeling may contribute not only to local invasion but also to treatment resistance.

Stromal remodeling is strongly implicated in prostate cancer progression, but the specific contribution of biological aging remains incompletely defined. Human studies support associations among reactive stroma, CAF-rich regions, ECM remodeling, and aggressive disease, whereas preclinical models provide mechanistic evidence that fibroblasts, matrix stiffness, proteases, and stromal-immune crosstalk can influence invasion and therapeutic response. Future work should distinguish aging-induced stromal changes from tumor-induced CAF programs using age-stratified human specimens, spatial profiling, matrix imaging, and functional models. Together, ECM remodeling provides an important link between stromal aging, inflammation, invasion, immune evasion, and therapy resistance [30,31,32,59,60,61,108,109,110,111,112,113,121,122,123].

### 4.3. Endothelial Aging and Angiogenesis

The vascular compartment is an essential component of the prostate tumor microenvironment. Endothelial cells regulate oxygen and nutrient delivery, immune-cell trafficking, tissue perfusion, angiogenesis, vascular permeability, and responses to therapy. During aging, endothelial cells undergo functional decline characterized by oxidative stress, mitochondrial dysfunction, impaired nitric oxide signaling, increased inflammatory activation, altered barrier integrity, reduced regenerative capacity, and senescence-associated changes [64,65]. These vascular changes may influence prostate tissue homeostasis and tumor progression.

Spatial and single-cell studies also highlight endothelial and perivascular heterogeneity in prostate cancer, suggesting that vascular aging should be evaluated as a niche-specific process involving endothelial cells, pericytes, immune-cell trafficking, and stromal organization rather than as a uniform decline in endothelial function [80].

Endothelial aging can contribute to chronic inflammation through increased expression of adhesion molecules, inflammatory cytokines, chemokines, and pro-thrombotic mediators. These changes may facilitate recruitment of immune cells into tissues but may also promote persistent inflammatory activation and vascular dysfunction. In the prostate tumor microenvironment, altered endothelial function may affect the composition and localization of immune-cell infiltrates, including T cells, macrophages, monocytes, and neutrophils. Thus, vascular aging may indirectly shape tumor immunity by regulating immune-cell access and inflammatory trafficking [33,34,35,37,38,39,64,65].

Angiogenesis is required for tumor growth beyond a limited size and contributes to progression by supplying oxygen, nutrients, and routes for dissemination. Prostate cancer angiogenesis is regulated by tumor cells, stromal fibroblasts, macrophages, endothelial cells, hypoxia, inflammatory cytokines, and growth factors such as VEGF [62,63,66]. Aging-associated inflammation and senescence-associated secretory programs can increase production of pro-angiogenic mediators, including VEGF, IL-6, IL-8/CXCL8, and matrix-remodeling enzymes. These factors may support abnormal vascular remodeling within the tumor microenvironment.

However, tumor-associated vasculature is often structurally and functionally abnormal. Tumor vessels may be tortuous, leaky, poorly organized, and inefficient at oxygen delivery. This can produce hypoxic regions that activate HIF-1α and other stress-response pathways [63,67]. Hypoxia can promote angiogenesis, metabolic adaptation, immune suppression, ECM remodeling, and resistance to therapy. In aged tissues, pre-existing vascular dysfunction may further intensify hypoxic stress and inflammatory signaling, thereby contributing to tumor progression.

Endothelial senescence may also influence therapeutic response. Impaired vascular function can affect drug delivery, radiation response, immune-cell infiltration, and tissue repair after therapy. Radiation and chemotherapy can themselves induce endothelial stress or senescence, potentially amplifying inflammatory tissue injury [64,65,66,67]. In older patients, baseline vascular aging and comorbidities such as obesity, diabetes, hypertension, and metabolic syndrome may further modify treatment tolerance and tumor response. Although these relationships require additional investigation in prostate cancer, they highlight the importance of considering vascular aging as part of the tumor microenvironment.

The vascular compartment also interacts closely with stromal and immune cells. Perivascular macrophages, fibroblasts, pericytes, and endothelial cells can form specialized niches that regulate immune-cell entry, matrix remodeling, angiogenesis, and tumor-cell survival. In aging tissues, these perivascular niches may be altered by chronic inflammation, senescence, oxidative stress, and metabolic dysfunction. This may affect not only local tumor growth but also the ability of tumor cells to invade surrounding tissues and access vascular routes for dissemination [30,31,64,65,66,67].

Vascular remodeling in prostate cancer is complex and may depend on tumor stage, stromal context, endothelial state, immune-cell trafficking, and host factors such as aging and metabolic comorbidity. Aging may be one underappreciated host factor that influences vascular remodeling. Single-cell and spatial transcriptomic analyses highlight endothelial and perivascular heterogeneity, supporting the need to evaluate vascular aging as a niche-specific process rather than a uniform decline in endothelial function. Overall, endothelial aging and angiogenesis remain incompletely understood components of the aging prostate tumor microenvironment, but they may contribute to hypoxia, inflammatory trafficking, altered perfusion, immune modulation, and therapy response [66,67].

#### Section Summary

Stromal aging is a major component of the prostate tumor microenvironment. Aging fibroblasts, senescent stromal cells, CAFs, remodeled ECM, and dysfunctional endothelial cells can collectively alter epithelial behavior, immune-cell trafficking, tissue mechanics, angiogenesis, and therapeutic response. These stromal changes may cooperate with cellular senescence and immune aging to create a chronic inflammatory niche that supports prostate cancer progression. Understanding stromal aging will be essential for defining biomarkers and therapeutic strategies that target the aging tumor microenvironment without disrupting normal tissue repair and homeostasis (Table 3).

## 5. Metabolic Stress and Inflammatory Metabolism in Aging-Associated Prostate Cancer

### 5.1. Mitochondrial Dysfunction and Oxidative Stress

Metabolic stress is a major feature of biological aging and is closely linked to chronic inflammation. Among aging-associated metabolic alterations, mitochondrial dysfunction and oxidative stress are particularly important because they can influence epithelial homeostasis, immune-cell activity, stromal remodeling, and tumor progression. Mitochondria regulate ATP production, redox balance, apoptosis, calcium signaling, innate immune activation, and biosynthetic metabolism. With aging, mitochondrial function may decline because of accumulated mitochondrial DNA damage, impaired mitophagy, altered mitochondrial dynamics, reduced respiratory efficiency, and increased production of ROS [6,75]. These changes can generate persistent cellular stress and promote inflammatory signaling within tissues.

ROS can have both physiological and pathological roles. At moderate levels, ROS participate in normal signaling, host defense, and tissue adaptation. However, excessive or persistent ROS can damage DNA, proteins, lipids, and organelles, thereby promoting genomic instability, senescence, and inflammatory activation [75,76]. In the prostate, chronic oxidative stress may contribute to epithelial injury, stromal dysfunction, immune-cell recruitment, and altered tissue repair. Oxidative DNA damage may also cooperate with age-associated decline in DNA repair capacity to increase mutational burden or cellular stress responses.

Mitochondrial dysfunction can promote inflammaging through several mechanisms. Damaged mitochondria can release mitochondrial DNA, cardiolipin, and other mitochondrial danger-associated molecular patterns that activate innate immune pathways, including cGAS–STING, inflammasome signaling, and NF-κB-associated inflammatory programs [75,82,83]. Impaired mitophagy may further increase accumulation of dysfunctional mitochondria and sustain inflammatory activation. These mechanisms link metabolic aging to chronic sterile inflammation and may contribute to the inflammatory prostate tumor microenvironment.

In prostate cancer, mitochondrial metabolism is complex and context-dependent. Normal prostate epithelial cells have unique metabolic features related to citrate production and secretion, whereas malignant transformation is associated with altered energy metabolism, lipid biosynthesis, oxidative phosphorylation, and redox regulation [69,72,115]. During cancer progression, tumor cells may adapt their mitochondrial function to support survival, proliferation, stress resistance, and response to therapy. Aging-associated mitochondrial dysfunction in stromal and immune cells may further influence the tumor microenvironment by altering cytokine production, immune-cell polarization, and tissue remodeling.

Oxidative stress can also interact with cellular senescence. Persistent ROS can induce senescence through DNA damage response activation, p53–p21^CIP1^ signaling, p16^INK4a^–RB pathway activation, and mitochondrial stress responses. Senescent cells, in turn, can produce additional ROS and inflammatory mediators, generating a feed-forward loop between oxidative stress, senescence, and chronic inflammation [16,20,75,82,83]. In the aging prostate microenvironment, this loop may involve epithelial cells, fibroblasts, endothelial cells, and immune cells.

Mitochondrial dysfunction may also influence immune-cell function. T cells, macrophages, dendritic cells, and myeloid-derived suppressor cells rely on metabolic programming to regulate activation, differentiation, effector function, and immune suppression. Aging-associated mitochondrial stress may impair cytotoxic T-cell activity, alter macrophage inflammatory states, reduce antigen presentation, and promote immune exhaustion-like phenotypes [25,26,27,28,75,114,115,116,117]. Therefore, mitochondrial aging may contribute to prostate cancer progression not only through effects on epithelial cells but also through remodeling of the immune microenvironment.

Therapeutically, mitochondrial stress and oxidative inflammation represent potential intervention points, but broad antioxidant strategies have shown inconsistent benefits in cancer prevention and treatment, likely because ROS can have both tumor-suppressive and tumor-promoting functions. More precise approaches that restore mitochondrial quality control, improve mitophagy, modulate redox-sensitive inflammatory pathways, or reduce harmful senescence-associated oxidative stress may be more relevant. In prostate cancer, future cell-type-specific studies will be needed to define whether mitochondrial dysfunction arises mainly from tumor epithelium, immune cells, fibroblasts, endothelial cells, or distinct aging-associated niches [75,76,98,99,100,101,102,103,104].

### 5.2. Obesity, Aging, and Systemic Inflammation

Obesity frequently coexists with aging and can amplify systemic inflammation, metabolic dysfunction, and cancer-related risk. Although aging and obesity are biologically distinct, they share several inflammatory and metabolic features, including adipose tissue inflammation, insulin resistance, altered lipid metabolism, mitochondrial stress, oxidative injury, immune-cell dysfunction, and increased circulating cytokines [68,125,126,127,128,129]. These overlapping processes may converge in older men to create a systemic environment that modifies prostate cancer progression.

Adipose tissue is an active endocrine and immune organ. During obesity, adipocytes can become hypertrophic and stressed, leading to altered secretion of adipokines, cytokines, free fatty acids, and metabolic mediators. Obese adipose tissue often contains increased macrophage infiltration, inflammatory cytokine production, hypoxia, fibrosis, and impaired metabolic regulation [68,74,125]. These changes contribute to chronic low-grade inflammation and may affect distant organs, including the prostate. In older individuals, age-associated immune remodeling, cellular senescence, and impaired inflammatory resolution may further intensify obesity-related inflammatory signaling.

Insulin resistance and altered insulin/IGF signaling are important metabolic consequences of obesity and aging. Hyperinsulinemia and increased IGF pathway activity may influence cell growth, survival, metabolism, and inflammatory signaling [68,69,74,125,126,127,128,129]. In prostate cancer, insulin/IGF-related pathways have been studied in relation to cancer risk, progression, and treatment response. Although findings across epidemiological and mechanistic studies are not always uniform, metabolic syndrome and obesity-associated endocrine changes may contribute to a tumor-supportive environment in at least some patient subsets.

Mechanistic studies further support crosstalk among obesity, insulin signaling, and IL-17-related inflammation. In obese mouse models of prostate cancer, hyperinsulinemia enhanced IL-17-induced downstream pro-inflammatory gene expression, increased IL-17 receptor A levels, and promoted development of more invasive prostate cancer. This effect was linked to insulin/IGF1-mediated suppression of GSK3 activity, which reduced GSK3-mediated phosphorylation and degradation of IL-17 receptor and thereby strengthened IL-17 signaling [130,131]. In a related metabolic-inflammation study, IL-17A differentially induced inflammatory and metabolic gene expression in adipose tissues from lean and obese mice, including genes related to cytokine production and metabolic regulation [130]. Together, these findings suggest that obesity may not simply increase systemic inflammatory tone but may also alter tissue responsiveness to inflammatory cytokines such as IL-17.

Obesity can also alter sex hormone metabolism, adipokine balance, and systemic inflammatory tone. Leptin, adiponectin, resistin, and other adipose-derived factors can influence immune-cell function, inflammation, angiogenesis, and cancer-cell behavior [68,74,127]. Increased leptin and reduced adiponectin, often observed in obesity, may favor inflammatory and growth-promoting signaling. In prostate cancer, the biological effects of adipokines are likely context-dependent and may vary according to tumor stage, androgen status, metabolic health, and local tissue environment.

The relationship between obesity and prostate cancer is clinically complex. Some studies suggest that obesity is associated with more aggressive disease, higher risk of biochemical recurrence, increased prostate cancer mortality, and worse treatment outcomes, whereas associations with overall prostate cancer incidence are less consistent [68,125,126,127,128,129]. This inconsistency likely reflects several confounders. BMI does not distinguish visceral adiposity, sarcopenic obesity, metabolic health, insulin resistance, or systemic inflammatory burden. Detection bias, PSA hemodilution, prostate size, race/ethnicity, comorbidities, treatment selection, and androgen deprivation therapy-induced metabolic changes may also modify observed associations. Therefore, future studies should move beyond BMI alone and incorporate metabolic health phenotypes, body composition, inflammatory biomarkers, insulin/IGF status, adipokine profiles, and ancestry-informed analyses.

In the aging prostate microenvironment, obesity-associated systemic inflammation may interact with local senescence, stromal remodeling, myeloid activation, T-cell dysfunction, endothelial changes, and metabolic stress. Circulating cytokines, adipokines, insulin/IGF signals, free fatty acids, and lipid mediators may affect prostate epithelial, immune, endothelial, and stromal cells, whereas tumor-derived factors may also influence systemic metabolism. Overall, obesity should be framed as a broad inflammatory and metabolic modifier of aging-associated prostate cancer rather than as a uniform risk factor. Future studies should distinguish systemic metabolic risk, local tumor metabolic adaptation, and aging-associated metabolic inflammation using body composition, metabolic health, ancestry-informed analyses, and inflammatory biomarkers [68,74,125,126,127,128,129].

### 5.3. Lipid Metabolism and Inflammatory Signaling

Lipid metabolism is central to both prostate biology and immune regulation. Lipids serve as structural components of membranes, energy substrates, signaling molecules, and regulators of inflammation. Prostate cancer cells frequently display altered lipid uptake, de novo lipogenesis, fatty acid oxidation, cholesterol metabolism, and membrane lipid remodeling [69,70,71,72,73,132,133,134,135]. These metabolic changes can support cell growth, survival, androgen receptor signaling, membrane synthesis, oxidative stress adaptation, and therapy resistance.

Aging can alter lipid metabolism at systemic and tissue levels. Age-associated changes may include increased adiposity, altered fatty acid handling, impaired mitochondrial β-oxidation, changes in circulating lipoproteins, increased lipid peroxidation, and accumulation of bioactive lipid mediators [6,25,69,70,71,72,73,74,75,84,114]. These changes may contribute to chronic inflammation and metabolic dysfunction. In the prostate microenvironment, lipid dysregulation may influence epithelial cells, immune cells, stromal fibroblasts, and endothelial cells.

Bioactive lipids can regulate inflammatory signaling. Eicosanoids, prostaglandins, leukotrienes, sphingolipids, oxidized lipids, lysophospholipids, bile acids, and fatty acid derivatives can modulate immune-cell activation, cytokine production, macrophage polarization, T-cell differentiation, vascular function, and tissue remodeling [70,71,72,73]. Some lipid mediators promote inflammation, whereas others participate in resolution of inflammation. Therefore, the balance between pro-inflammatory and pro-resolving lipid signals may be important in determining whether lipid metabolism supports tissue repair or chronic inflammation.

Lipid metabolism also affects immune-cell function. T cells require metabolic reprogramming during activation and differentiation, and lipid availability can influence effector T-cell function, regulatory T-cell activity, exhaustion-like states, and memory formation [70,71,72,73]. Macrophages and myeloid-derived suppressor cells are also highly sensitive to lipid metabolism. Lipid accumulation, fatty acid oxidation, cholesterol handling, and oxidized lipid signaling can promote inflammatory or immunosuppressive myeloid phenotypes depending on context [70,71,72,73]. In aging and obesity, altered lipid availability may therefore reshape the prostate immune microenvironment.

In prostate cancer cells, lipid metabolic pathways may interact with oncogenic and hormonal signaling. Androgen receptor activity has been linked to regulation of lipid synthesis and metabolic gene expression, and prostate tumors may rely on lipid metabolic adaptation during progression or therapeutic stress [71,132,133,134,135]. Changes in cholesterol metabolism and fatty acid synthesis may also influence membrane composition, intracellular signaling, and survival pathways. Although these mechanisms are not exclusive to aging, they may be modified by age-associated systemic metabolic changes.

Lipid peroxidation and oxidative lipid damage may further connect metabolic stress to inflammation. Oxidized lipids can act as danger signals, activate pattern-recognition receptors, influence macrophage function, and promote tissue injury [75,76]. In aged tissues, increased oxidative stress may enhance lipid peroxidation and generate inflammatory lipid products. These processes may contribute to senescence, stromal remodeling, endothelial dysfunction, and immune activation in the prostate microenvironment.

Because lipid metabolism is highly complex, specific lipid species may have different effects depending on concentration, cell type, receptor expression, enzymatic metabolism, disease stage, and tissue location. Lipid dysregulation should therefore be viewed as one component of metabolic inflammaging rather than as a single dominant mechanism. Integrating lipidomics, metabolomics, isotope tracing, spatial metabolite imaging, and cell-type-specific profiling may help distinguish systemic metabolic inflammation from local tumor metabolic adaptation in the aging prostate tumor microenvironment [69,70,71,72,73].

### 5.4. Nutrient-Sensing Pathways

Nutrient-sensing pathways integrate information about energy availability, growth factors, amino acids, glucose, oxygen, and cellular stress. These pathways are central regulators of aging, metabolism, inflammation, and cancer [6,8,69,72]. In the aging prostate microenvironment, altered nutrient sensing may influence epithelial cell growth, immune-cell function, senescence, stromal remodeling, and therapeutic response.

The mechanistic target of rapamycin pathway is one of the best-characterized nutrient-sensing systems. mTOR integrates signals from amino acids, growth factors, oxygen, and energy status to regulate protein synthesis, autophagy, lipid metabolism, mitochondrial function, and cell growth [6,69,95,134]. Increased or dysregulated mTOR activity has been associated with aging, cellular senescence, inflammatory signaling, and cancer progression. In senescent cells, mTOR can contribute to SASP production, and mTOR inhibition has been explored as a strategy to modulate aging-associated inflammation. In prostate cancer, PI3K–AKT–mTOR signaling is particularly relevant because PTEN loss and pathway activation are common events in tumor progression.

AMP-activated protein kinase is another major metabolic regulator. AMPK senses cellular energy stress and promotes catabolic processes that restore energy balance while inhibiting anabolic growth pathways [69,72,74]. AMPK activation can suppress mTOR signaling, promote autophagy, improve mitochondrial homeostasis, and reduce inflammatory signaling in some contexts. Because aging and metabolic disease are associated with altered energy sensing, AMPK-related pathways may influence both prostate epithelial biology and immune-cell function. Metformin and exercise-associated metabolic effects have often been discussed in relation to AMPK activation, although their clinical impact in prostate cancer remains an active area of investigation.

Insulin and IGF signaling link systemic metabolic state to cellular growth and survival. These pathways can activate PI3K–AKT–mTOR, MAPK, and other downstream signaling networks [68,69,74,125,126,127,128,129]. In obesity and metabolic syndrome, hyperinsulinemia and altered IGF signaling may contribute to inflammatory and growth-promoting environments. In prostate cancer, insulin/IGF-related pathways may interact with androgen receptor signaling, tumor metabolism, and treatment response. However, the relationship is complex and may depend on tumor genotype, metabolic status, and therapy context.

Nuclear receptors also play important roles in metabolic inflammation. Peroxisome proliferator-activated receptors regulate lipid metabolism, adipogenesis, insulin sensitivity, macrophage function, and inflammatory responses [69,70,71,72,73,74]. Liver X receptors, farnesoid X receptors, and other lipid-sensing nuclear receptors can influence cholesterol metabolism, bile acid signaling, immune-cell activity, and tissue inflammation. In the aging prostate microenvironment, nuclear receptor pathways may provide links between systemic metabolism, immune-cell function, and local inflammatory signaling.

Autophagy is closely connected to nutrient sensing, mitochondrial quality control, senescence, and immune regulation. Aging is often associated with impaired autophagy, which can lead to accumulation of damaged organelles, increased oxidative stress, altered antigen presentation, and inflammatory activation [6,8,69,72]. In cancer, autophagy has context-dependent roles. It can suppress tumor initiation by maintaining cellular homeostasis, but established tumors may use autophagy to survive metabolic stress and therapy. In prostate cancer, autophagy may influence tumor-cell survival, androgen deprivation response, immune-cell function, and senescence-associated inflammation.

These nutrient-sensing pathways interact extensively with each other [69,70,71,72,73,74,75,76]. mTOR, AMPK, insulin/IGF signaling, nuclear receptors, and autophagy form interconnected networks that regulate growth, metabolism, inflammation, and stress adaptation. Aging and obesity can shift these networks toward chronic inflammatory and tumor-supportive states. However, because these pathways also maintain normal tissue function, therapeutic targeting requires careful balance. For example, broad suppression of mTOR or inflammatory metabolism may have different effects in tumor cells, immune cells, stromal cells, and normal tissues.

The relationship between metabolic stress and prostate cancer is biologically plausible but clinically complex. Obesity, insulin resistance, mitochondrial dysfunction, lipid dysregulation, altered nutrient sensing, and oxidative stress can promote inflammatory and tumor-supportive pathways in preclinical models, but clinical associations with prostate cancer incidence, progression, and mortality remain variable. Future work should define how mTOR, AMPK, insulin/IGF signaling, nuclear receptors, and autophagy operate in specific epithelial, stromal, immune, and endothelial populations, while distinguishing systemic metabolic risk from local tumor metabolic adaptation and aging-associated metabolic inflammation [69,70,71,72,73,74,75,76].

#### Section Summary

Metabolic stress is an important component of aging-associated prostate cancer biology. Mitochondrial dysfunction, oxidative stress, obesity-associated inflammation, lipid dysregulation, and altered nutrient-sensing can interact with senescence, immune aging, stromal activation, vascular dysfunction, and therapeutic stress. Integrating metabolomics, lipidomics, spatial profiling, and cell-type-specific functional studies will be important for identifying safe strategies to target metabolic inflammaging (Table 4).

## 6. Consequences of Inflammaging for Prostate Cancer Progression and Therapy Response

Inflammaging can influence prostate cancer progression through multiple interconnected mechanisms rather than through a single pathway. Senescent cells, immune dysfunction, myeloid remodeling, stromal activation, ECM remodeling, vascular dysfunction, and metabolic stress may converge to alter tumor-cell behavior and therapeutic response. These processes can affect early tumor initiation, local invasion, immune evasion, treatment resistance, and adaptive tumor-cell states. This multicellular crosstalk is summarized conceptually in Figure 1 and discussed in detail below.

### 6.1. Tumor Initiation and Early Progression

Aging-associated inflammaging may influence prostate cancer development from the earliest stages of epithelial transformation. The aged prostate microenvironment is characterized by chronic inflammatory signaling, cellular senescence, oxidative stress, stromal remodeling, immune dysfunction, and altered tissue repair. These changes may create a permissive environment in which genetically or epigenetically altered epithelial cells are more likely to survive, expand, and interact with tumor-supportive stromal and immune compartments [6,8,9,10,11,12,13,29,92,93,94,136]. Although prostate cancer initiation is driven in part by tumor-intrinsic alterations, the surrounding aged tissue environment may determine whether early lesions remain indolent or progress toward clinically significant disease.

Chronic inflammation can promote early tumorigenesis through several mechanisms. Inflammatory cytokines and ROS can induce DNA damage, epigenetic alterations, and stress-response signaling in epithelial cells. Senescent epithelial and stromal cells can produce SASP factors that alter local growth control, recruit immune cells, and remodel ECM. Inflammatory immune cells can release cytokines, chemokines, proteases, and growth factors that support tissue remodeling and epithelial adaptation. Together, these processes may disturb normal epithelial homeostasis and increase the likelihood that premalignant cells acquire growth advantages [16,20,21,22,23,40,41,42,43,44,45,46,47,53,54,55,92,93,94].

The prostate is particularly sensitive to stromal–epithelial communication [56,57,58,106,107,108]. During normal development and adult homeostasis, stromal cells provide signals that regulate epithelial differentiation, androgen responsiveness, and tissue architecture. Aging can disrupt these regulatory interactions. Senescent or activated fibroblasts may produce inflammatory and growth-promoting mediators, while remodeled ECM may alter epithelial polarity, adhesion, and mechanotransduction. These stromal changes may cooperate with epithelial alterations to support early tumor expansion.

Immune surveillance is also critical during early tumor development [25,26,27,28,33,34,35,114,115,116,117]. In younger or immunologically intact tissues, emerging abnormal cells may be recognized and eliminated by cytotoxic T cells, natural killer cells, macrophages, and antigen-presenting cells. Aging may impair these protective responses through reduced antigen presentation, diminished cytotoxic function, T-cell exhaustion-like features, and myeloid-mediated immune suppression [26,27,28,33,35]. Thus, immune aging may allow abnormal epithelial cells to persist while chronic inflammatory signaling simultaneously promotes tissue remodeling.

Inflammaging may also influence early prostate cancer progression by shifting the local consequences of senescence. As discussed above, the biological outcome of senescence depends on timing, cell type, persistence, and immune clearance. In aged tissue, impaired clearance and persistent SASP activity may allow senescence-associated programs to support neighboring non-senescent epithelial or stromal cells, thereby favoring early tumor expansion [14,15,18,20,21,24,88,89,90,91,92,93,94].

Therefore, prostate tumor initiation and early progression should be considered not only as cell-intrinsic genetic events but also as tissue-level processes influenced by aging. The aged microenvironment may provide inflammatory, stromal, metabolic, and immune signals that increase the probability of tumor expansion. This concept highlights the importance of age-appropriate experimental models and human studies that examine both tumor cells and the surrounding aging tissue context [77,78,79].

### 6.2. Local Invasion and Tumor Progression

As prostate cancer progresses, tumor cells interact more extensively with stromal fibroblasts, immune cells, endothelial cells, ECM, and soluble inflammatory mediators [30,31,32,109,110,111,112,113]. Inflammaging may contribute to local invasion and disease progression by promoting matrix remodeling, fibroblast activation, immune-cell recruitment, angiogenesis, and stress-adaptive signaling. These processes may allow tumor cells to breach normal tissue boundaries, invade surrounding stroma, and adapt to hostile microenvironmental conditions.

ECM remodeling is a major mechanism through which inflammaging may support invasion. Senescent stromal cells, cancer-associated fibroblasts, macrophages, and inflammatory epithelial cells can produce MMPs and other proteases that degrade basement membrane and interstitial matrix components [56]. This remodeling can create physical paths for tumor-cell movement and release matrix-bound growth factors. Changes in collagen deposition, fibronectin expression, crosslinking, and tissue stiffness can also activate mechanotransduction pathways that promote migration, survival, and invasive behavior.

Inflammatory cytokines can directly affect tumor-cell behavior. IL-6, TNF-α, IL-1 family cytokines, IL-17-related signaling, TGF-β, and chemokines may activate pathways such as NF-κB, STAT3, MAPK, SMAD, and PI3K–AKT signaling [30,31,40,41,42,43,44,45,46,47,53,54,55]. These pathways can regulate survival, motility, stress tolerance, and interaction with stromal cells. Although the effects of individual cytokines are context-dependent, persistent exposure to inflammatory mediators may help tumor cells adapt to the aging tissue environment.

Myeloid cells are particularly important in invasion-supportive inflammatory niches. Tumor-associated macrophages, inflammatory monocytes, neutrophils, and myeloid-derived suppressor cells can produce cytokines, chemokines, angiogenic mediators, proteases, and ROS [30,32,33,34,35,44]. These cells may support matrix remodeling, tumor-cell survival, vascular remodeling, and immune suppression. Aging-associated myeloid remodeling may therefore amplify tumor-promoting inflammation and support local progression.

Stromal fibroblasts also contribute to local invasion [30,31,56,57,58]. Aging fibroblasts and cancer-associated fibroblasts can produce growth factors, cytokines, ECM proteins, and matrix-remodeling enzymes that alter epithelial behavior. Fibroblast-derived factors may increase epithelial proliferation, motility, survival, and resistance to stress. In addition, fibroblast-mediated matrix remodeling can increase tissue stiffness and create invasion-supportive tracks within the tumor microenvironment.

Endothelial aging and angiogenesis may further support tumor progression. Abnormal vasculature can create hypoxic regions that activate HIF-1α and other stress-response pathways. Hypoxia can promote angiogenesis, inflammatory signaling, metabolic adaptation, and resistance to therapy [62,63,64,65,66,67]. In aged tissues, pre-existing vascular dysfunction may worsen hypoxia and impair immune-cell trafficking, thereby contributing to tumor progression and treatment resistance.

Importantly, local invasion is not caused by inflammation alone. Tumor-intrinsic alterations, androgen receptor signaling changes, tumor suppressor loss, epigenetic remodeling, and therapeutic pressure all contribute to prostate cancer progression [4,135,137,138,139,140,141,142]. However, inflammaging may provide a permissive microenvironment that supports the consequences of these tumor-intrinsic changes. This tissue-level perspective is important for understanding why similar genetic alterations may lead to different clinical outcomes depending on host age, immune status, metabolic health, and stromal context.

### 6.3. Immune Evasion and Treatment Resistance

Immune evasion is a central feature of cancer progression and may be strongly influenced by aging. The aged immune system is often characterized by reduced naïve T-cell diversity, impaired cytotoxic function, altered antigen presentation, myeloid skewing, chronic inflammatory activation, and increased immune-suppressive signaling [25,26,27,28,29,33,34,35,37,38,39,114,115,116,117]. These changes can weaken anti-tumor immunity while allowing inflammatory programs that support tumor survival and adaptation.

In prostate cancer, immune evasion can involve multiple mechanisms. Tumors may reduce antigen presentation, recruit suppressive myeloid cells, expand regulatory T-cell activity, exclude effector T cells, increase checkpoint ligand expression, and establish cytokine environments that impair cytotoxic function. Aging may intensify these mechanisms by reducing immune-cell plasticity and promoting chronic suppressive or exhausted immune states. As a result, older tumor microenvironments may contain immune cells that are present but functionally ineffective.

Myeloid-mediated immune suppression is especially relevant. Macrophages and myeloid-derived suppressor cells can inhibit T-cell activation and function through arginine depletion, ROS, nitric oxide, IL-10, TGF-β, prostaglandins, and checkpoint-related pathways [30,32,33,34,35,37,38,39,44]. These suppressive myeloid populations can also promote angiogenesis, tissue remodeling, and resistance to therapy. Aging-associated myeloid skewing may therefore contribute simultaneously to immune evasion and tumor progression.

T-cell dysfunction may also limit therapeutic response. Aging and chronic antigen exposure can increase exhaustion-like features in T cells, including impaired proliferation, reduced cytokine production, and altered expression of inhibitory receptors. In prostate cancer, limited responsiveness to immune checkpoint blockade in unselected patients may reflect multiple barriers, including low tumor immunogenicity, immunosuppressive myeloid cells, stromal exclusion, and insufficient effector T-cell function [33,34,35,37,38,39,143,144,145,146,147]. Inflammaging may add another layer of resistance by creating a chronically inflamed yet poorly cytotoxic immune environment.

Mechanistically, immune checkpoint resistance in prostate cancer is best understood as a multicellular niche problem rather than failure of a single checkpoint pathway. The major barriers include limited tumor immunogenicity in many tumors, weak effector T-cell infiltration, suppressive myeloid and Treg activity, stromal/ECM-mediated immune exclusion, androgen-regulated immune programs, and lack of robust predictive biomarkers in unselected patients. From an inflammaging perspective, chronic inflammation may intensify this paradox by increasing cytokine and myeloid signaling without generating effective cytotoxic immunity. Therefore, strategies to improve immunotherapy response in prostate cancer may need to combine checkpoint modulation with approaches that reprogram myeloid cells, improve antigen presentation, reduce stromal exclusion, or identify biomarker-defined inflammatory niches.

Inflammatory signaling can also affect response to androgen receptor-directed therapies, radiation, chemotherapy, and targeted treatments. Cytokines such as IL-6, TNF-α, TGF-β, and IL-17-related mediators can activate survival and stress-response pathways that may help tumor cells withstand therapy [84,85,96,97,101,102,103,104]. Treatment-induced senescence may contribute to growth arrest, but persistent senescent tumor or stromal cells can also reshape the local microenvironment through SASP activity. Thus, as outlined in Section 2.3, the therapeutic consequence of senescence depends on whether senescent cells are transiently induced and cleared or persist as a source of inflammatory adaptation.

A prostate cancer-specific example is the treatment-induced secretory response of prostate cancer-associated stromal cells [148]. DNA-damaging therapies can induce stromal stress responses and secreted factors, including WNT16B and inflammatory mediators, that promote prostate cancer cell survival and resistance to therapy. This work provides direct evidence that therapy-altered stromal secretory programs in human prostate cancer-associated tissue can influence treatment response, supporting the relevance of SASP-like and damage-associated secretory programs in prostate cancer-specific therapeutic resistance.

Stromal and vascular aging may further modify treatment response. Dense ECM can impair drug penetration and immune-cell infiltration. Hypoxia can reduce radiation sensitivity and promote adaptive stress responses. Endothelial dysfunction can alter perfusion and tissue repair. Metabolic dysfunction can influence both tumor-cell survival and immune-cell activity. Therefore, therapy resistance in aging-associated prostate cancer may arise from interactions among tumor cells, senescent cells, immune populations, stromal architecture, vasculature, and systemic metabolic status [59,60,61,62,63,64,65,66,67,69,72,121,122,123].

These observations suggest that effective treatment of prostate cancer in older patients may require more than targeting tumor cells alone. Therapeutic strategies that modulate inflammatory cytokines, suppress harmful SASP activity, reprogram myeloid cells, improve immune surveillance, normalize stromal barriers, or correct metabolic inflammation may enhance response to conventional therapies [33,34,35,37,38,39,98,99,100,101,102,103,104,143,144,145,146,147]. However, these approaches require careful development because inflammation and senescence can also have protective roles in tissue repair and tumor suppression.

### 6.4. Phenotypic Adaptation Under Inflammatory and Therapeutic Stress

Prostate cancer cells can adapt to changing microenvironmental and therapeutic conditions by altering signaling pathways, differentiation state, metabolic programs, and stress-response mechanisms [16,20,21,22,23,24,84,85,96,97,101]. Chronic inflammatory signaling may contribute to this adaptation by activating pathways involved in survival, repair, motility, immune interaction, and treatment tolerance. In the aging prostate microenvironment, persistent cytokine exposure, oxidative stress, stromal remodeling, hypoxia, and metabolic dysfunction may collectively create selective pressures that favor more adaptable tumor-cell states.

Inflammatory pathways such as NF-κB, STAT3, MAPK, PI3K–AKT, and TGF-β/SMAD signaling can regulate cell survival, stress adaptation, and epithelial behavior [30,69,82,83,95,107,108,134]. These pathways can be activated by cytokines, chemokines, growth factors, oxidative stress, matrix signaling, and therapy-induced injury. Persistent activation may allow tumor cells to survive inflammatory stress and adapt to treatment. However, the effects of these pathways are highly context-dependent and may differ according to tumor genotype, stage, androgen receptor status, immune composition, and treatment history.

Therapeutic stress can also modify the tumor microenvironment. Androgen deprivation therapy, androgen receptor pathway inhibitors, radiation, chemotherapy, and other treatments can induce cellular stress, inflammation, senescence, vascular changes, and immune remodeling [38,84,85,96,97,101,102,103,104]. These therapy-induced changes may enhance tumor-cell death or immune recognition, but in aged tissue they may also interact with pre-existing inflammaging to create residual inflammatory niches that support surviving tumor cells.

Chronic inflammation has been associated with altered epithelial differentiation, survival signaling, migratory behavior, and treatment response in multiple cancers. In prostate cancer, inflammatory and stromal signals may contribute to disease progression and resistance, but the precise mechanisms linking aging-associated inflammation to specific aggressive phenotypes remain under active investigation. Thus, inflammaging is best viewed as a permissive tissue context that supports tumor-cell adaptation rather than as a single deterministic pathway [4,33,34,35,37,38,39,137,138,139,140,141,142].

Future studies should determine how inflammatory and therapeutic stress interact in aged prostate tissues [4,35,39,137,138,139,140,141,142]. Key questions include which cell types produce therapy-induced inflammatory mediators, how senescent cells persist or are cleared after treatment, how aged immune cells respond to tumor injury, and whether biological aging markers predict treatment response. Answering these questions may help identify patients whose tumors are shaped by inflammaging and may benefit from microenvironment-targeted therapeutic strategies [33,34,35,98,99,100,101,102,103,104,143,144,145,146,147].

#### Section Summary

Inflammaging may influence prostate cancer initiation, local invasion, immune evasion, therapy resistance, and tumor-cell adaptation. These effects arise from interactions among senescent cells, cytokines, immune dysfunction, myeloid remodeling, stromal activation, ECM remodeling, vascular dysfunction, metabolic stress, and tumor-intrinsic alterations. Defining these multicellular interactions will be essential for biomarkers and interventions targeting aging-associated prostate cancer progression.

## 7. Therapeutic Implications of Targeting Inflammaging in Prostate Cancer

### 7.1. Cytokine-Targeted Approaches

Inflammaging is characterized by persistent cytokine and chemokine signaling; therefore, inflammatory pathways represent potential therapeutic targets in aging-associated prostate cancer. Cytokines such as IL-6, IL-17, IL-23, TNF-α, IL-1 family members, and TGF-β can influence immune-cell recruitment, stromal remodeling, epithelial stress responses, angiogenesis, and treatment resistance [33,34,35,40,41,42,43,44,45,46,47,53,54,55]. However, cytokine-targeted therapy should not be viewed as broad anti-inflammatory treatment. The goal is to identify dominant tumor-promoting inflammatory circuits while preserving protective immunity, host defense, and tissue repair.

Several cytokine pathways are particularly relevant. IL-6/JAK/STAT3 signaling can support tumor-cell survival, inflammatory adaptation, angiogenesis, androgen receptor pathway interactions, and therapy resistance [30,31,33,34,35,37,38,39,95]. IL-17-/IL-23-related signaling may be relevant in prostate tumors with Th17-associated inflammation, myeloid remodeling, or IL-23/IL-23R pathway activity, but its context-dependent immune functions require biomarker-guided evaluation [40,41,42,43,44,45,46,47,55]. TNF-α, IL-1, and NF-κB-related pathways can amplify cytokine cascades, chemokine production, and myeloid recruitment, whereas TGF-β can promote stromal remodeling, immune exclusion, and invasive behavior in selected contexts [9,10,12,30,82,83,95,107,108,111,113]. Because these pathways also regulate normal immune and repair functions, clinical translation will require pathway-specific biomarkers, careful toxicity monitoring, and rational combination strategies.

### 7.2. Senescence-Directed Therapies

Cellular senescence is a major contributor to aging-associated tissue inflammation, making senescence-directed therapy an attractive strategy for modifying the aged tumor microenvironment. Two broad approaches have emerged: senolytics, which aim to eliminate senescent cells, and senomorphics, which aim to suppress harmful SASP programs without necessarily killing senescent cells [98,99,100,101,102,103,104]. These strategies may be relevant to prostate cancer because senescent epithelial, stromal, endothelial, immune, or therapy-induced tumor cells may contribute to chronic inflammatory signaling and tissue remodeling.

A major challenge is that senescence is context-dependent. Acute tumor-cell senescence may restrict malignant transformation or contribute to treatment response, whereas persistent senescent cells can sustain SASP-driven inflammation, stromal activation, immune dysfunction, and therapy resistance [14,15,18,20,88,89,90,91,92,93,94]. Therefore, timing and cell type are critical. In prostate cancer, senescence-directed therapy may be most rational when persistent senescent cells remain after therapy or accumulate in aged stromal or immune compartments, rather than during early tumor-suppressive senescence.

Biomarker development will be essential. Potential biomarkers include p16^INK4a^, p21^CIP1^, DNA damage markers, senescence-associated β-galactosidase activity, SASP cytokines, senescence-related transcriptomic signatures, and spatial localization of senescent cells [14,15,19,39,84,85,96,97]. Because no single marker definitively identifies senescence in all contexts, combined tissue, spatial, and circulating biomarker panels will likely be needed before senolytic or senomorphic strategies can be safely tested in prostate cancer.

### 7.3. Metabolic and Lifestyle Interventions

Metabolic stress is closely linked to inflammaging, and interventions that improve metabolic health may reduce chronic inflammatory burden. In older men, obesity, insulin resistance, metabolic syndrome, mitochondrial dysfunction, lipid dysregulation, and systemic inflammation may influence prostate cancer biology [68,69,70,71,72,73,74,75,76,125,126,127,128,129,132,134,135]. Exercise, weight management, and dietary approaches may improve insulin sensitivity, adipose inflammation, mitochondrial function, vascular health, immune-cell activity, and treatment tolerance. These interventions should be viewed as supportive strategies that may modify the systemic inflammatory context rather than as stand-alone prostate cancer therapies.

Pharmacologic metabolic modulators are also of interest. Metformin has been studied because of its effects on insulin sensitivity, AMPK activation, mitochondrial metabolism, and inflammatory signaling [69,72]. Nutrient-sensing pathways such as mTOR, AMPK, insulin/IGF signaling, PPARs, and autophagy may also connect metabolic aging to senescence, immune remodeling, and tumor-cell adaptation [6,9,70,73,96,145]. However, these pathways function in tumor cells, immune cells, stromal cells, and normal tissues, making systemic targeting complex.

Future metabolic intervention studies should move beyond BMI alone. Patients may differ in visceral adiposity, sarcopenic obesity, metabolic health, insulin resistance, adipokine profiles, inflammatory burden, and androgen deprivation therapy-induced metabolic changes. Integrating metabolic phenotyping with tissue immune profiling, circulating cytokines, metabolomics, lipidomics, and treatment outcomes may help identify patients most likely to benefit from metabolic inflammaging-directed strategies.

### 7.4. Biomarker-Guided Patient Stratification

A major challenge in targeting inflammaging is patient heterogeneity. Chronological age alone is unlikely to identify patients with biologically aged or inflamed prostate tumor microenvironments [4,35,39,137,138,139,140,141,142]. Two men of the same age may differ substantially in senescent-cell burden, immune function, stromal remodeling, vascular health, systemic inflammation, metabolic status, comorbidities, and treatment tolerance. Therefore, biomarker-guided stratification will be essential for translating inflammaging biology into prostate cancer prevention and therapy.

Potential biomarkers include circulating cytokines and chemokines, SASP factors, immune-cell subsets, Th17/Treg balance, myeloid signatures, senescence-associated markers, metabolic indicators, and tissue-level inflammatory signatures [4,35,39,137,138,139,140,141,142]. Tissue-based technologies such as multiplex immunofluorescence, spatial transcriptomics, single-cell RNA sequencing, proteomics, and metabolomics may help determine whether inflammatory signals arise from senescent stromal cells, myeloid cells, T-cell subsets, endothelial cells, tumor cells, or adjacent benign tissue.

Ultimately, useful biomarkers should do more than measure inflammation. They should identify which component of inflammaging is dominant, whether it is therapeutically actionable, and whether targeting it is likely to improve clinical outcome. For example, IL-6/STAT3-high tumors, SASP-enriched tumors, myeloid-rich immunosuppressive tumors, and metabolically inflamed tumors may require different therapeutic strategies. Prospective validation will be necessary before such biomarkers can guide clinical decision-making.

### 7.5. Combination Strategies and Clinical Translation

Because inflammaging involves multiple interacting cell types and pathways, single-agent targeting may be insufficient in many cases. Rational combinations may include cytokine modulation with androgen receptor-directed therapy, senescence-directed therapy after radiation or systemic therapy, metabolic intervention with immune modulation, or stromal-targeted approaches with conventional treatment [33,34,35,37,38,39,98,99,100,101,102,103,104,143,144,145,146,147]. The goal should be precise modulation of harmful aging-associated inflammatory circuits rather than broad suppression of inflammation.

Safety and timing are central translational concerns. Inflammatory and senescence-associated pathways can promote tumor progression, but they also support host defense, wound healing, tissue repair, and tumor suppression. For example, cytokine blockade, JAK/STAT inhibition, immune-checkpoint combinations, senescence-directed therapies, and stromal/ECM-targeted approaches may carry risks related to infection, immune toxicity, cytopenias, thrombosis, impaired repair, or loss of protective senescence. Therefore, inflammaging-targeted combinations should be tested with careful attention to patient selection, biological age, comorbidities, functional reserve, and pathway-specific biomarkers.

Age-appropriate preclinical and clinical models will be essential for translation. Many prostate cancer studies use young animals that do not reflect the aged immune, stromal, vascular, and metabolic environments of human disease [77,78]. Future studies should test inflammaging-targeted strategies in aged models, obesity-associated models, senescence-enriched settings, organoids, explants, co-culture systems, and spatially profiled human specimens. Clinical trials should incorporate biological aging measures, metabolic health, inflammatory biomarkers, immune status, and functional reserve, rather than relying on chronological age.

### 7.6. Current Clinical Trial Landscape and Translational Challenges

Although inflammaging-related pathways provide a strong biological rationale for therapeutic intervention, current clinical evidence in prostate cancer remains limited. Available studies suggest that cytokine modulation, metabolic intervention, and JAK/STAT-pathway targeting should not be broadly applied to unselected patients but instead require biomarker-guided trial design. Representative completed and ongoing trials are summarized in Table 5.

Negative or modest trial results are informative. The MAST trial suggests that metabolic intervention should not be translated from broad epidemiologic or preclinical rationale alone; insulin resistance, obesity phenotype, inflammatory status, and metabolic biomarkers may be needed to identify responsive subgroups [149]. Similarly, early IL-23 blockade with tildrakizumab plus abiraterone was feasible but did not show objective responses in a small, unselected ARPI-resistant mCRPC cohort, supporting the need for pathway-specific biomarkers of IL-23/IL-23R activity [150]. The ongoing ruxolitinib plus enzalutamide trial will help determine whether JAK/STAT-pathway modulation has activity in mCRPC, but this approach remains investigational [151].

Future inflammaging-related trials should incorporate patient selection based on biological rather than chronological age alone. Useful stratification variables may include circulating cytokines, immune-cell states, senescence-associated markers, insulin/IGF status, metabolic health, myeloid signatures, and spatial immune-stromal features. Such designs may help distinguish true lack of therapeutic efficacy from failure to select the appropriate inflammaging-defined patient population.

#### Section Summary

**Figure 2:**
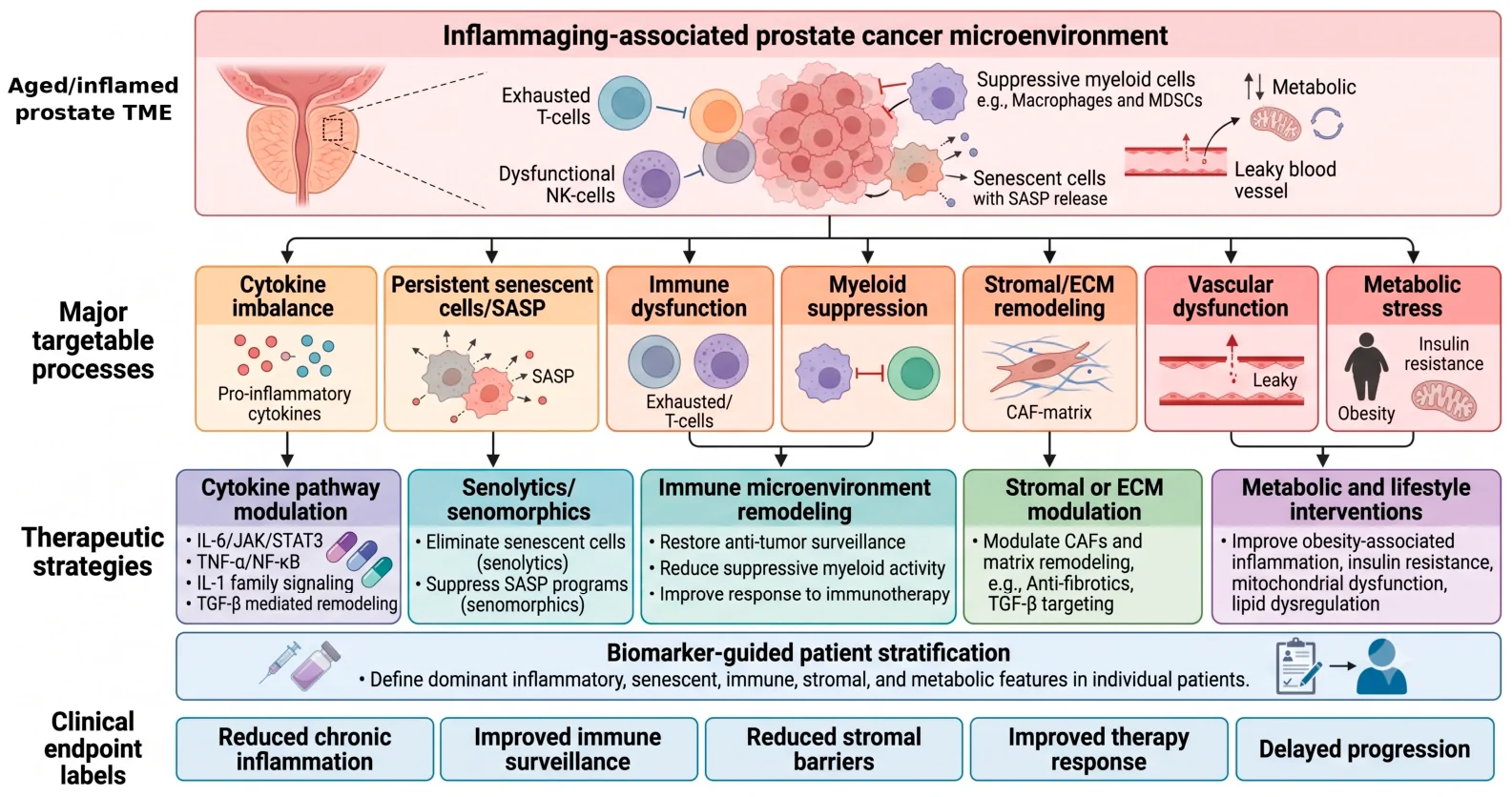
Therapeutic opportunities for targeting inflammaging-associated prostate cancer progression. The figure summarizes an aged/inflamed prostate tumor microenvironment characterized by dysfunctional T/NK cells, suppressive myeloid cells, senescent cells with SASP release, leaky vasculature, and metabolic stress. Major targetable processes include cytokine imbalance, persistent senescent cells/SASP, immune dysfunction, myeloid suppression, stromal/ECM remodeling, vascular dysfunction, and metabolic stress. Potential strategies include cytokine-pathway modulation, senolytics or senomorphics, immune microenvironment remodeling, stromal/ECM modulation, metabolic and lifestyle interventions, and biomarker-guided patient stratification. Arrows indicate proposed links between the aged/inflamed tumor microenvironment, targetable processes, therapeutic strategies, and clinical endpoint labels; inhibitory bars indicate suppressive immune interactions. These approaches aim to reduce chronic inflammation, improve immune surveillance, reduce stromal barriers, improve therapeutic response, and potentially delay progression. Selected key references: [33,35,55,98,101,102,143,147].

Targeting inflammaging offers therapeutic opportunities in prostate cancer, including cytokine modulation, senescence-directed therapy, metabolic intervention, immune remodeling, stromal modulation, and biomarker-guided treatment selection. However, these approaches remain largely investigational and must balance suppression of harmful chronic inflammation with preservation of protective immunity, tissue repair, and tumor-suppressive senescence. Representative clinical trials and broader therapeutic strategies are summarized in Table 5 and Table 6, respectively, and the therapeutic framework is shown in Figure 2.

## 8. Knowledge Gaps and Future Directions

### 8.1. Distinguishing Chronological Age from Biological Aging

A major challenge in studying aging-associated prostate cancer is distinguishing chronological age from biological aging. Chronological age is easy to measure, but it does not fully capture the molecular, cellular, immune, metabolic, vascular, and functional changes that occur during aging. Individuals of the same chronological age may differ substantially in senescent-cell burden, SASP activity, immune dysfunction, stromal remodeling, mitochondrial stress, metabolic inflammation, vascular aging, and tissue repair capacity [6,8,9,25,26,27,28,29,114,115,116,117].

Biological aging biomarkers may help identify patients whose tumors develop within biologically aged or inflamed microenvironments. Candidate markers include epigenetic aging clocks, senescence-associated markers, inflammatory cytokine profiles, immune-cell composition, metabolic signatures, mitochondrial stress markers, and tissue-level stromal or vascular remodeling features. In prostate cancer, these markers should be integrated with tumor grade, stage, genomic alterations, treatment history, comorbidities, and functional reserve.

Distinguishing chronological from biological aging may improve both mechanistic research and clinical translation. Older patients with low inflammatory burden and preserved immune function may differ substantially from younger patients with obesity-associated inflammation, insulin resistance, high senescent-cell burden, or metabolic dysfunction. Therefore, future studies should treat aging as a measurable biological state rather than as a simple demographic covariate.

### 8.2. Need for Age-Appropriate Experimental Models

Many preclinical prostate cancer studies rely on young animals or rapidly growing tumor models. While these systems are useful for defining tumor-intrinsic mechanisms, they may not represent the aged immune, stromal, vascular, senescent, and metabolic environments in which human prostate cancer most commonly develops. This limitation is especially important for studies of inflammaging, immune evasion, senescence-directed therapy, and metabolic intervention.

Age-defined models are needed to determine how host age modifies tumor initiation, progression, immune remodeling, and treatment response. A spatially and temporally controlled Pten-null prostate cancer mouse model generated at different adult ages provides evidence that host age can accelerate PI3K/AKT/mTOR signaling and prostate cancer onset and progression [77]. Similar approaches should incorporate host age, tumor genotype, immune status, stromal context, metabolic state, and treatment timing.

Functional validation remains essential. Single-cell, spatial, and omics approaches can identify age-associated signatures, but these signatures are not sufficient to establish causality. Future studies should combine age-defined animal models with patient-derived organoids, tissue explants, stromal-epithelial co-cultures, immune-cell co-cultures, and three-dimensional ECM systems that incorporate aged stromal cells, aged immune cells, senescent-cell components, or metabolic stress conditions.

### 8.3. Defining Cell-Type-Specific and Spatial Mechanisms

Inflammaging is a multicellular process, and a major unresolved question is which cell types initiate, maintain, and respond to aging-associated inflammatory signals in prostate cancer. Senescent epithelial cells, stromal fibroblasts, macrophages, myeloid-derived suppressor cells, T-cell subsets, endothelial cells, adipose-associated cells, and tumor cells may all contribute to the inflammatory tumor microenvironment. Their relative contributions likely vary by disease stage, tumor genotype, treatment exposure, metabolic status, and host age.

Recent single-cell and spatial studies have shown that prostate cancer-associated immune, stromal, endothelial, and epithelial compartments are composed of diverse cell states rather than uniform populations [80,120,152,153]. These approaches can identify exhausted T-cell states, immunosuppressive Treg activity, heterogeneous macrophage and myeloid populations, angiogenic endothelial programs, CAF-like states, and spatially organized inflammatory niches. However, aging-associated inflammaging should be mapped not only to broad cell labels, but also to cell states, spatial position, ligand-receptor interactions, and functional relationships with nearby tumor epithelium.

Spatial information is particularly important because cell proximity often determines biological function. For example, inflammatory myeloid cells near tumor epithelium may have different effects from those located in stromal or perivascular regions. Similarly, senescent stromal cells adjacent to tumor glands may influence epithelial behavior differently from senescent cells in surrounding benign tissue. Future studies should integrate single-cell RNA sequencing, spatial transcriptomics, multiplex immunofluorescence, imaging mass cytometry, proteomics, metabolomics, lipidomics, and functional validation to define clinically meaningful inflammaging niches.

### 8.4. Clinical Heterogeneity: Race/Ethnicity, Metabolic Health, and Microbiome Interactions

Clinical heterogeneity is a major barrier to translating inflammaging biology into prostate cancer care. Race and ethnicity, genetic ancestry, structural determinants of health, access to screening and treatment, environmental exposures, diet, obesity, metabolic health, comorbidities, and treatment history may all influence prostate cancer outcomes. In the United States, Black men have higher prostate cancer incidence and mortality than other racial and ethnic groups, but these disparities should not be interpreted as purely biological; they likely reflect complex interactions among ancestry-related biology, social determinants, access to care, comorbidities, tumor biology, and treatment patterns [154]. Future inflammaging studies should therefore include adequately powered and ancestry-informed cohorts rather than extrapolating from populations that are not representative of prostate cancer disparities.

Metabolic health is another underdeveloped area. BMI alone does not distinguish visceral adiposity, sarcopenic obesity, insulin resistance, adipokine imbalance, systemic inflammation, or androgen deprivation therapy-induced metabolic changes. Because obesity and aging may converge on IL-17-related inflammation, insulin/IGF signaling, myeloid activation, lipid dysregulation, and immune dysfunction, future studies should integrate body composition, metabolic biomarkers, inflammatory markers, treatment status, and tissue profiling.

Microbiome interactions also require deeper study. Prostate, urinary, gut, and intratumoral microbial communities may influence inflammation, immune tone, androgen metabolism, treatment response, and systemic metabolic signaling [30,155,156]. However, microbiome studies are vulnerable to contamination, antibiotic exposure, diet, sampling method, sequencing platform, and population differences. Future studies should combine microbiome profiling with metabolomics, immune profiling, spatial tissue analysis, and age-stratified clinical data to determine whether microbiome–inflammaging interactions are causal, correlative, or therapeutically actionable.

### 8.5. MicroRNAs and Extracellular Vesicle-Mediated Communication

Small non-coding RNAs, particularly microRNAs, represent an emerging regulatory layer in senescence-associated inflammation, but direct evidence linking specific microRNAs to aging-specific inflammaging in human prostate tumors remains limited. Therefore, microRNAs may be best viewed as a future direction rather than an established mechanism in aging-associated prostate cancer.

A particularly important area is extracellular vesicle-mediated transfer of microRNAs and other regulatory molecules among senescent epithelial cells, stromal fibroblasts, endothelial cells, immune cells, and tumor cells. Senescent cells can release extracellular vesicles that may transmit inflammatory, metabolic, or stress-associated signals to neighboring cells, potentially shaping SASP activity, immune recruitment, stromal remodeling, and therapy response. Future studies should integrate EV profiling, microRNA sequencing, single-cell transcriptomics, spatial proteomics, and age-stratified prostate cancer cohorts to determine whether EV-associated microRNAs define senescent-cell communication networks in the aging prostate tumor microenvironment [157,158].

### 8.6. Lessons from Senolytic and Geroscience Trials Outside Prostate Cancer

Senolytic and geroscience trials outside prostate cancer provide useful lessons for translation. Early studies of dasatinib plus quercetin in idiopathic pulmonary fibrosis and diabetic kidney disease suggest that senolytic strategies can be tested in humans and may affect physical function or senescent-cell burden in selected disease contexts [159,160]. However, these studies were small, disease-specific, and not designed to establish prostate cancer efficacy.

These experiences highlight several principles relevant to prostate cancer. First, senescence-directed therapy requires biomarkers that identify senescent-cell burden, cell type, tissue location, and SASP activity. Second, timing matters: eliminating senescent cells too early may interfere with tumor-suppressive senescence or tissue repair, whereas targeting persistent senescent cells after therapy may be more rational. Third, safety is essential, particularly in older patients with comorbidities, impaired wound healing, immune vulnerability, or cardiovascular risk.

For prostate cancer, senolytic or senomorphic strategies should therefore be evaluated cautiously and in biomarker-defined settings. Future studies should determine whether senescent cells are located primarily in tumor epithelium, stromal fibroblasts, endothelial cells, immune cells, or therapy-exposed tissue, and whether their removal or modulation improves treatment response without compromising protective senescence, host defense, or tissue repair.

### 8.7. Future Conceptual Framework

Future studies should view prostate cancer as a disease shaped by both tumor-intrinsic alterations and host tissue aging. Biological aging may create permissive prostate microenvironments through senescence, SASP activity, immune dysfunction, stromal remodeling, vascular aging, metabolic stress, microbiome-associated inflammation, and impaired tissue repair. These processes may generate distinct inflammaging phenotypes, such as senescent stromal inflammation, myeloid suppression, metabolic inflammation, vascular dysfunction, ECM remodeling, or microbiome-associated immune changes. Defining these phenotypes may support biomarker development and guide age-aware prevention or therapeutic strategies.

#### Section Summary

Major knowledge gaps remain in understanding how aging-associated inflammation shapes prostate cancer. Future studies should distinguish chronological from biological aging, use age-appropriate models, define cell-type-specific and spatial mechanisms, address clinical heterogeneity, incorporate race/ethnicity and metabolic health, investigate microbiome interactions, and evaluate microRNA/EV communication and senescence-directed strategies cautiously. Addressing these gaps may reveal new approaches to delay aggressive prostate cancer progression and improve treatment selection in older men.

## 9. Conclusions

Prostate cancer is strongly associated with aging, but the biological relationship between aging and prostate cancer progression extends beyond chronological time and accumulated genetic alterations. Aging reshapes the prostate tissue environment through cellular senescence, chronic low-grade inflammation, immune dysfunction, stromal remodeling, ECM alteration, vascular changes, metabolic stress, and impaired tissue repair [4,6,8,9]. Together, these processes create an inflammaging-associated microenvironment that may influence tumor initiation, local progression, immune evasion, therapeutic response, and disease outcome.

Cellular senescence is a central component of this aging-associated microenvironment, but its biological effects are context-dependent. Transient senescence may restrict damaged cells and support tumor suppression, whereas persistent senescent epithelial, stromal, endothelial, and immune cells may sustain SASP-driven inflammation, ECM remodeling, immune-cell recruitment, vascular dysfunction, and impaired tissue repair. Thus, the consequences of senescence in prostate cancer depend on timing, cell type, immune clearance, and tissue context.

Immune, stromal, and metabolic aging further cooperate to shape the prostate tumor microenvironment. Age-associated changes in T-cell diversity, Th17/Treg balance, IL-17/IL-23-related inflammation, myeloid-cell activity, antigen presentation, and NF-κB/STAT3 signaling may create an immune state that is chronically inflamed but not effectively tumoricidal. At the same time, aging fibroblasts, cancer-associated fibroblasts, remodeled ECM, endothelial dysfunction, mitochondrial stress, oxidative injury, obesity-associated inflammation, lipid dysregulation, and altered nutrient-sensing pathways may promote tumor growth, invasion, immune-cell trafficking, angiogenesis, and therapy resistance. These mechanisms should be viewed as interconnected components of a multicellular aging network rather than isolated drivers.

Therapeutically, inflammaging presents both opportunities and challenges. Cytokine-targeted approaches, senescence-directed therapies, metabolic interventions, stromal modulation, myeloid reprogramming, and biomarker-guided immune strategies may eventually help reduce harmful aging-associated inflammation in selected patients. However, inflammation and senescence can also support immune defense, tissue repair, and tumor suppression. Therefore, future strategies should aim to modulate maladaptive chronic inflammation while preserving protective immune and tissue-regenerative functions.

Future progress will require distinguishing chronological age from biological aging and defining clinically meaningful inflammaging phenotypes. Integrated biomarkers combining tissue profiling, circulating inflammatory markers, immune signatures, senescence markers, metabolomic or lipidomic features, and spatial organization may improve risk stratification and guide therapeutic selection. Age-appropriate experimental models, human aging-stratified cohorts, single-cell and spatial technologies, functional co-culture systems, and multi-omics approaches will be essential for identifying which components of inflammaging are protective, harmful, or therapeutically actionable. By viewing aging as an active biological context rather than a passive demographic variable, the field may uncover new strategies to delay aggressive prostate cancer progression while preserving immune defense, tissue repair, and tumor-suppressive senescence.

## Figures and Tables

**Table 1:** Context-dependent consequences of senescence in prostate cancer (selected key references: [13,14,15,84,92,98]).

Senescence Context	Major Cell Types	Potential Protective Effect	Potential Tumor-Promoting Effect	Key Implication
Acute oncogene- or damage-induced senescence	Premalignant epithelial cells	Growth arrest, tumor suppression, immune-mediated clearance of damaged cells	May be bypassed during progression if tumor-suppressor pathways are lost or reprogrammed	Senescence can act as an early barrier to malignant transformation
Persistent epithelial senescence	Prostate epithelial or tumor-adjacent epithelial cells	Restriction of damaged-cell proliferation	SASP-mediated inflammation, epithelial stress, altered stromal and immune signaling	Persistent epithelial senescence may shift from protective arrest to inflammatory remodeling
Stromal fibroblast senescence	Fibroblasts, smooth muscle-associated stromal cells	Transient tissue-repair response after injury	SASP, growth factors, MMPs, ECM remodeling, paracrine epithelial support	Aged stromal senescence may create tumor-supportive niches
CAF-like stromal activation with senescence overlap	Cancer-associated fibroblasts, activated stromal cells	Possible wound-repair or matrix-restoration functions in acute settings	Matrix stiffening, immune modulation, stromal barriers, invasion-supportive remodeling	Senescent fibroblasts and CAFs are not identical but may cooperate in aged tumors
Endothelial senescence	Endothelial cells, pericyte-associated vascular niches	Acute vascular stress response	Vascular dysfunction, altered permeability, hypoxia, impaired immune-cell trafficking	Vascular aging may influence immune access, angiogenesis, and therapy response
Immune-cell senescence or exhaustion-like states	T cells, myeloid cells, antigen-presenting cells	Limits excessive immune activation in some contexts	Reduced immune surveillance, impaired clearance of senescent cells, chronic inflammatory signaling	Immune aging can combine weak tumor killing with persistent inflammation
Therapy-induced senescence	Tumor cells, stromal cells, endothelial cells after radiation/systemic therapy	Initial growth arrest and treatment response	Persistent SASP, residual inflammatory niches, relapse-supportive adaptation	Timing is critical; persistent senescent cells may be more targetable than acute senescence

**Table 2:** Immune-aging mechanisms relevant to prostate cancer (selected key references: [25,27,35,40,44,114]).

Immune-Aging Feature	Major Cell Types	Representative Mediators/Pathways	Potential Prostate Cancer Relevance
Reduced immune surveillance	CD8^+^ T cells, dendritic cells, NK cells	Exhaustion markers, impaired antigen presentation, reduced cytotoxicity	Tumor persistence, immune escape
Chronic cytokine imbalance	T cells, macrophages, stromal cells, senescent cells	IL-6, TNF-α, IL-1, IL-17, IL-23, TGF-β	Inflammation, stromal remodeling, therapy resistance
Th17/Treg imbalance	CD4^+^ T cells	IL-17, IL-23, FOXP3, IL-10, TGF-β	Chronic inflammation and immune suppression
Myeloid remodeling	Macrophages, MDSCs, monocytes, dendritic cells	IL-6, IL-10, TGF-β, IL-23, CCL2, CXCL chemokines	Immune suppression, angiogenesis, matrix remodeling
Altered immune transcriptional states	T cells, myeloid cells	NF-κB, STAT3, RORγt, FOXP3, BATF, IRF family factors	Persistent inflammatory or suppressive immune programs

**Table 3:** Stromal aging mechanisms in prostate cancer (selected key references: [30,56,58,59,64,107]).

Stromal Feature	Major Cell Type/Component	Key Mediators	Potential Consequence
Senescent fibroblast activation	Fibroblasts, smooth muscle cells	SASP-related inflammatory and matrix-remodeling signals	Chronic inflammation, paracrine epithelial support
CAF remodeling	Cancer-associated fibroblasts	Growth factors, cytokines, ECM proteins	Tumor growth, invasion, immune modulation
ECM stiffness/remodeling	Collagen, fibronectin, basement membrane	Integrins, FAK/SRC, YAP/TAZ, MMPs	Migration, invasion, treatment resistance
Vascular aging	Endothelial cells, pericytes	VEGF, HIF-1α, ROS, adhesion molecules	Hypoxia, altered immune trafficking, angiogenesis
Stromal–immune crosstalk	Fibroblasts, macrophages, T cells	CCL2, CXCL chemokines, TGF-β, IL-6	Immune suppression, inflammatory niche formation

**Table 4:** Metabolic stress pathways in aging-associated prostate cancer (selected key references: [6,68,69,72,75]).

Metabolic Feature	Major Mechanisms	Potential Inflammatory Effect	Possible Prostate Cancer Relevance
Mitochondrial dysfunction	mtDNA damage, impaired mitophagy, altered respiration	ROS, cGAS–STING, inflammasome activation	Senescence, inflammation, therapy response
Oxidative stress	Excess ROS, lipid peroxidation, DNA damage	NF-κB activation, tissue injury, SASP amplification	Epithelial stress, stromal remodeling
Obesity-associated inflammation	Adipose inflammation, insulin resistance, adipokine imbalance	Systemic cytokines, myeloid activation	Tumor progression, immune dysfunction
Lipid dysregulation	Altered fatty acids, cholesterol, oxidized lipids, eicosanoids	Immune-cell modulation, inflammatory lipid signaling	Tumor metabolism, immune remodeling
Nutrient-sensing changes	mTOR, AMPK, insulin/IGF, PPARs, autophagy	Altered SASP, immune metabolism, stress adaptation	Growth signaling, metabolic adaptation, resistance

**Table 5:** Representative clinical trials targeting inflammaging-related pathways in prostate cancer (selected key references: [149,150,151]).

Strategy/Pathway	Trial/Intervention	Phase/Status	Population	Key Endpoint/Result	Main Translational Lesson
Metabolic intervention	Metformin Active Surveillance Trial; NCT01864096 [149]	Phase III, completed	Low-risk localized prostate cancer on active surveillance	Randomized placebo-controlled trial; 408 patients; no significant improvement in progression-free survival	Metabolic modulation requires metabolic stratification
IL-23 blockade	Tildrakizumab plus abiraterone acetate; NCT04458311 [150]	Phase I/II; phase I reported	ARPI-resistant mCRPC	Well tolerated, but no objective responses; median PFS 3.7 months; median OS 9.7 months	Requires biomarker-selected IL-23/IL-23R-active patients
JAK/STAT pathway targeting	Ruxolitinib plus enzalutamide; NCT06616155 [151]	Phase I/II, ongoing	mCRPC	Safety/best dose; PSA50 and/or RECIST response within 6 months	Investigational; needs toxicity and biomarker monitoring

**Table 6:** Therapeutic strategies targeting inflammaging in prostate cancer (selected key references: [33,35,55,98,101,102,143,147]).

Therapeutic Strategy	Main Target	Rationale	Potential Benefit	Major Caution
Cytokine modulation	IL-6/JAK/STAT3; IL-17/IL-23; TNF-α/NF-κB; IL-1; TGF-β	Chronic cytokine signaling can drive inflammation, stromal remodeling, immune suppression, and resistance	May reduce protumor inflammation and improve treatment response	Clinical benefit in prostate cancer remains unproven; IL-23 blockade may increase infection risk; JAK/STAT inhibition may cause cytopenias, infection risk, or thrombotic concerns
Senolytics	Persistent senescent cells	Senescent cells can sustain SASP-driven inflammation	May reduce senescent-cell burden and chronic inflammatory niches	Not established in prostate cancer; senescence can be protective; timing, cell type, and drug-specific toxicities are major concerns
Senomorphics	SASP pathways (mTOR, JAK/STAT, p38 MAPK, NF-κB, cGAS–STING)	Suppress harmful SASP without eliminating all senescent cells	May preserve growth arrest while reducing inflammatory remodeling	Optimal targets and patient selection remain unclear
Metabolic/lifestyle intervention	Obesity, insulin resistance, mitochondrial dysfunction, lipid dysregulation	Metabolic stress can amplify inflammaging and alter immune/stromal function	May improve systemic inflammation, metabolic health, and treatment tolerance	Supportive rather than stand-alone therapy; metformin trials have shown mixed or negative oncologic results; age-specific benefit remains unclear
Immune/myeloid remodeling	Myeloid cells, T-cell dysfunction, antigen presentation, immune checkpoints	Aged tumor microenvironments may be inflamed but poorly tumoricidal	May improve immune surveillance and immunotherapy response	Checkpoint blockade has limited activity in unselected prostate cancer and may cause immune-related adverse events; biomarker selection is essential
Stromal/ECM modulation	CAFs, ECM stiffness, TGF-β-related remodeling, matrix proteases	Stromal remodeling can promote invasion, immune exclusion, hypoxia, and drug resistance	May improve immune-cell infiltration, drug delivery, and tissue organization	Broad ECM/MMP targeting has failed in prior cancer trials; specificity, toxicity, and wound-healing effects are concerns
Vascular normalization	Endothelial dysfunction, angiogenesis, hypoxia	Vascular dysfunction can impair immune trafficking and treatment delivery	May improve perfusion, oxygenation, immune access, and therapy response	Effects are context-dependent
Biomarker-guided stratification	Cytokines, SASP markers, immune signatures, metabolic profiles, spatial features	Inflammaging is heterogeneous and not present in all patients	May identify patients most likely to benefit from targeted strategies	Biomarkers still require clinical validation

## Data Availability

No new data were created or analyzed in this study. Data sharing is not applicable to this article.

## Abbreviations

The following abbreviations are used in this manuscript: AMPK, AMP-activated protein kinase; AP-1, activator protein 1; ARPI, androgen receptor pathway inhibitor; BATF, basic leucine zipper ATF-like transcription factor; CAFs, cancer-associated fibroblasts; CCL, C-C motif chemokine ligand; CD, cluster of differentiation; cGAS-STING, cyclic GMP-AMP synthase-stimulator of interferon genes; CTLA-4, cytotoxic T-lymphocyte-associated protein 4; CXCL, C-X-C motif chemokine ligand; DNA, deoxyribonucleic acid; ECM, extracellular matrix; EV, extracellular vesicle; FAK, focal adhesion kinase; FOXP3, forkhead box P3; GM-CSF, granulocyte-macrophage colony-stimulating factor; HIF-1α, hypoxia-inducible factor 1-alpha; IGF, insulin-like growth factor; IL, interleukin; IRF, interferon regulatory factor; JAK, Janus kinase; MAPK, mitogen-activated protein kinase; mCRPC, metastatic castration-resistant prostate cancer; MDSCs, myeloid-derived suppressor cells; MMPs, matrix metalloproteinases; mtDNA, mitochondrial DNA; mTOR, mechanistic target of rapamycin; NF-κB, nuclear factor kappa B; NK, natural killer; PI3K-AKT, phosphoinositide 3-kinase-protein kinase B; PPARs, peroxisome proliferator-activated receptors; PSA50, ≥50% decline in prostate-specific antigen; PTEN, phosphatase and tensin homolog; RB, retinoblastoma protein; RECIST, Response Evaluation Criteria in Solid Tumors; ROS, reactive oxygen species; SA-β-gal, senescence-associated β-galactosidase; SASP, senescence-associated secretory phenotype; SMAD, mothers against decapentaplegic homolog; STAT, signal transducer and activator of transcription; TGF-β, transforming growth factor beta; Th17, T helper 17; TME, tumor microenvironment; TNF-α, tumor necrosis factor alpha; Tregs, regulatory T cells; VEGF, vascular endothelial growth factor; YAP/TAZ, yes-associated protein/transcriptional coactivator with PDZ-binding motif.

## References

[1] Kratzer T.B., Mazzitelli N., Star J., Dahut W.L., Jemal A., Siegel R.L. (2025). Prostate cancer statistics, 2025. CA Cancer J. Clin..

[2] Surveillance, Epidemiology, and End Results Program, National Cancer Institute Cancer Stat Facts: Prostate Cancer. https://seer.cancer.gov/statfacts/html/prost.html.

[3] Siegel R.L., Kratzer T.B., Wagle N.S., Sung H., Jemal A. (2026). Cancer statistics, 2026. CA Cancer J. Clin..

[4] Rebello R.J., Oing C., Knudsen K.E., Loeb S., Johnson D.C., Reiter R.E., Gillessen S., Van der Kwast T., Bristow R.G. (2021). Prostate cancer. Nat. Rev. Dis. Prim..

[5] Kulkarni P.A., Cortés-Penfield N.W., Brehm T.J., Wagenlehner F., Gupta K., Leitner L., Trautner B.W. (2026). State-of-the-Art Review: Diagnosis and Management of Acute and Chronic Bacterial Prostatitis. Clin. Infect. Dis..

[6] López-Otín C., Blasco M.A., Partridge L., Serrano M., Kroemer G. (2023). Hallmarks of aging: An expanding universe. Cell.

[7] Bray F., Laversanne M., Sung H., Ferlay J., Siegel R.L., Soerjomataram I., Jemal A. (2024). Global cancer statistics 2022: GLOBOCAN estimates of incidence and mortality worldwide for 36 cancers in 185 countries. CA Cancer J. Clin..

[8] López-Otín C., Blasco M.A., Partridge L., Serrano M., Kroemer G. (2013). The hallmarks of aging. Cell.

[9] Franceschi C., Garagnani P., Parini P., Giuliani C., Santoro A. (2018). Inflammaging: A new immune-metabolic viewpoint for age-related diseases. Nat. Rev. Endocrinol..

[10] Franceschi C., Bonafè M., Valensin S., Olivieri F., De Luca M., Ottaviani E., De Benedictis G. (2000). Inflamm-aging. An evolutionary perspective on immunosenescence. Ann. N. Y. Acad. Sci..

[11] Fulop T., Larbi A., Dupuis G., Le Page A., Frost E.H., Cohen A.A., Witkowski J.M., Franceschi C. (2017). Immunosenescence and Inflamm-Aging As Two Sides of the Same Coin: Friends or Foes?. Front. Immunol..

[12] Furman D., Campisi J., Verdin E., Carrera-Bastos P., Targ S., Franceschi C., Ferrucci L., Gilroy D.W., Fasano A., Miller G.W. (2019). Chronic inflammation in the etiology of disease across the life span. Nat. Med..

[13] Campisi J. (2013). Aging, cellular senescence, and cancer. Annu. Rev. Physiol..

[14] Gorgoulis V., Adams P.D., Alimonti A., Bennett D.C., Bischof O., Bishop C., Campisi J., Collado M., Evangelou K., Ferbeyre G. (2019). Cellular Senescence: Defining a Path Forward. Cell.

[15] Childs B.G., Durik M., Baker D.J., van Deursen J.M. (2015). Cellular senescence in aging and age-related disease: From mechanisms to therapy. Nat. Med..

[16] Rodier F., Coppe J.P., Patil C.K., Hoeijmakers W.A., Munoz D.P., Raza S.R., Freund A., Campeau E., Davalos A.R., Campisi J. (2009). Persistent DNA damage signalling triggers senescence-associated inflammatory cytokine secretion. Nat. Cell Biol..

[17] Hayflick L., Moorhead P.S. (1961). The serial cultivation of human diploid cell strains. Exp. Cell Res..

[18] Serrano M., Lin A.W., McCurrach M.E., Beach D., Lowe S.W. (1997). Oncogenic ras provokes premature cell senescence associated with accumulation of p53 and p16INK4a. Cell.

[19] Dimri G.P., Lee X., Basile G., Acosta M., Scott G., Roskelley C., Medrano E.E., Linskens M., Rubelj I., Pereira-Smith O. (1995). A biomarker that identifies senescent human cells in culture and in aging skin in vivo. Proc. Natl. Acad. Sci. USA.

[20] Coppe J.P., Patil C.K., Rodier F., Sun Y., Munoz D.P., Goldstein J., Nelson P.S., Desprez P.Y., Campisi J. (2008). Senescence-associated secretory phenotypes reveal cell-nonautonomous functions of oncogenic RAS and the p53 tumor suppressor. PLoS Biol..

[21] Coppe J.P., Desprez P.Y., Krtolica A., Campisi J. (2010). The senescence-associated secretory phenotype: The dark side of tumor suppression. Annu. Rev. Pathol..

[22] Orjalo A.V., Bhaumik D., Gengler B.K., Scott G.K., Campisi J. (2009). Cell surface-bound IL-1alpha is an upstream regulator of the senescence-associated IL-6/IL-8 cytokine network. Proc. Natl. Acad. Sci. USA.

[23] Kuilman T., Michaloglou C., Vredeveld L.C., Douma S., van Doorn R., Desmet C.J., Aarden L.A., Mooi W.J., Peeper D.S. (2008). Oncogene-induced senescence relayed by an interleukin-dependent inflammatory network. Cell.

[24] Freund A., Orjalo A.V., Desprez P.Y., Campisi J. (2010). Inflammatory networks during cellular senescence: Causes and consequences. Trends Mol. Med..

[25] Nikolich-Zugich J. (2018). The twilight of immunity: Emerging concepts in aging of the immune system. Nat. Immunol..

[26] Liu Z., Liang Q., Ren Y., Guo C., Ge X., Wang L., Cheng Q., Luo P., Zhang Y., Han X. (2023). Immunosenescence: Molecular mechanisms and diseases. Signal Transduct. Target. Ther..

[27] Liu Q., Li J., Sun X., Lin J., Yu Z., Xiao Y., Li D., Sun B., Bao H., Liu Y. (2025). Immunosenescence and cancer: Molecular hallmarks, tumor microenvironment remodeling, and age-specific immunotherapy challenges. J. Hematol. Oncol..

[28] Yousefzadeh M.J., Flores R.R., Zhu Y., Schmiechen Z.C., Brooks R.W., Trussoni C.E., Cui Y., Angelini L., Lee K.A., McGowan S.J. (2021). An aged immune system drives senescence and ageing of solid organs. Nature.

[29] Fane M., Weeraratna A.T. (2020). How the ageing microenvironment influences tumour progression. Nat. Rev. Cancer.

[30] Chen L., Xu Y.X., Wang Y.S., Ren Y.Y., Dong X.M., Wu P., Xie T., Zhang Q., Zhou J.L. (2024). Prostate cancer microenvironment: Multidimensional regulation of immune cells, vascular system, stromal cells, and microbiota. Mol. Cancer.

[31] Pakula H., Pederzoli F., Fanelli G.N., Nuzzo P.V., Rodrigues S., Loda M. (2024). Deciphering the Tumor Microenvironment in Prostate Cancer: A Focus on the Stromal Component. Cancers.

[32] Gu Q., Qi A., Wang N., Zhou Z., Zhou X. (2024). Macrophage dynamics in prostate cancer: Molecular to therapeutic insights. Biomed. Pharmacother..

[33] Stultz J., Fong L. (2021). How to turn up the heat on the cold immune microenvironment of metastatic prostate cancer. Prostate Cancer Prostatic Dis..

[34] Huang J., Ojo A., Tsao S., Horowitz A., Kyprianou N., Tsao C.K. (2025). Overcoming Immune Evasion in the Prostate Tumor Microenvironment: Novel Targeted Strategies to Improve Treatment Outcomes. Cancers.

[35] Li M., Kwantwi L.B., Wang C., Xiao Q. (2025). Tumor microenvironment-mediated immune evasion and resistance in prostate cancer: Mechanisms, cross-talk, and therapeutic opportunities. Clin. Exp. Med..

[36] Salminen A. (2020). Activation of immunosuppressive network in the aging process. Ageing Res. Rev..

[37] Rastogi I., McNeel D.G. (2025). Prostate tumor immune microenvironment changes following immunotherapy shared by patients who developed anti-tumor response or immune-related adverse events. Oncoimmunology.

[38] Paal K., Stranz B., Thurner E.M., Niedrist T., Renner W., Langsenlehner T. (2024). Radiotherapy and inflammaging: The influence of prostate cancer radiotherapy on systemic inflammation. World J. Urol..

[39] Majewska Z., Zajkowska M., Paczek S., Nowinski A.R., Sokolska W., Gryko M., Orywal K. (2025). The Clinical Relevance of Tumor Biomarkers in Prostate Cancer-A Review. Cancers.

[40] Liu S., Rivero S.L., Zhang B., Shen K., Li Z., Niu T., Rowan B.G., Jazwinski S.M., Abdel-Mageed A.B., Steele C. (2024). BATF-dependent Th17 cells act through the IL-23R pathway to promote prostate adenocarcinoma initiation and progression. J. Natl. Cancer Inst..

[41] Zhang Q., Liu S., Xiong Z., Wang A.R., Myers L., Melamed J., Tang W.W., You Z. (2014). Interleukin-17 promotes development of castration-resistant prostate cancer potentially through creating an immunotolerant and pro-angiogenic tumor microenvironment. Prostate.

[42] Zhang Q., Liu S., Ge D., Cunningham D.M., Huang F., Ma L., Burris T.P., You Z. (2017). Targeting Th17-IL-17 Pathway in Prevention of Micro-Invasive Prostate Cancer in a Mouse Model. Prostate.

[43] Liu S., Liu F., Zhang B., Yan P., Rowan B.G., Abdel-Mageed A.B., Steele C., Jazwinski S.M., Moroz K., Norton E.B. (2020). CD4+ T helper 17 cell response of aged mice promotes prostate cancer cell migration and invasion. Prostate.

[44] Calcinotto A., Spataro C., Zagato E., Di Mitri D., Gil V., Crespo M., De Bernardis G., Losa M., Mirenda M., Pasquini E. (2018). IL-23 secreted by myeloid cells drives castration-resistant prostate cancer. Nature.

[45] Mirlekar B. (2022). Impact of IL-23 in prostate cancer. Asian J. Androl..

[46] Radej S., Szewc M., Maciejewski R. (2022). Prostate Infiltration by Treg and Th17 Cells as an Immune Response to *Propionibacterium acnes* Infection in the Course of Benign Prostatic Hyperplasia and Prostate Cancer. Int. J. Mol. Sci..

[47] Kiełb P., Kaczorowski M., Kowalczyk K., Piotrowska A., Nowak Ł., Krajewski W., Chorbińska J., Dudek K., Dzięgiel P., Hałoń A. (2023). Role of IL-17A and IL-17RA in Prostate Cancer with Lymph Nodes Metastasis: Expression Patterns and Clinical Significance. Cancers.

[48] Harrington L.E., Hatton R.D., Mangan P.R., Turner H., Murphy T.L., Murphy K.M., Weaver C.T. (2005). Interleukin 17-producing CD4+ effector T cells develop via a lineage distinct from the T helper type 1 and 2 lineages. Nat. Immunol..

[49] Park H., Li Z., Yang X.O., Chang S.H., Nurieva R., Wang Y.H., Wang Y., Hood L., Zhu Z., Tian Q. (2005). A distinct lineage of CD4 T cells regulates tissue inflammation by producing interleukin 17. Nat. Immunol..

[50] Ivanov I.I., McKenzie B.S., Zhou L., Tadokoro C.E., Lepelley A., Lafaille J.J., Cua D.J., Littman D.R. (2006). The orphan nuclear receptor RORgammat directs the differentiation program of proinflammatory IL-17+ T helper cells. Cell.

[51] Bettelli E., Carrier Y., Gao W., Korn T., Strom T.B., Oukka M., Weiner H.L., Kuchroo V.K. (2006). Reciprocal developmental pathways for the generation of pathogenic effector TH17 and regulatory T cells. Nature.

[52] Veldhoen M., Hocking R.J., Atkins C.J., Locksley R.M., Stockinger B. (2006). TGFbeta in the context of an inflammatory cytokine milieu supports de novo differentiation of IL-17-producing T cells. Immunity.

[53] Kryczek I., Banerjee M., Cheng P., Vatan L., Szeliga W., Wei S., Huang E., Finlayson E., Simeone D., Welling T.H. (2009). Phenotype, distribution, generation, and functional and clinical relevance of Th17 cells in the human tumor environments. Blood.

[54] Martin-Orozco N., Muranski P., Chung Y., Yang X.O., Yamazaki T., Lu S., Hwu P., Restifo N.P., Overwijk W.W., Dong C. (2009). T helper 17 cells promote cytotoxic T cell activation in tumor immunity. Immunity.

[55] Langowski J.L., Zhang X., Wu L., Mattson J.D., Chen T., Smith K., Basham B., McClanahan T., Kastelein R.A., Oft M. (2006). IL-23 promotes tumour incidence and growth. Nature.

[56] Tuxhorn J.A., Ayala G.E., Smith M.J., Smith V.C., Dang T.D., Rowley D.R. (2002). Reactive stroma in human prostate cancer: Induction of myofibroblast phenotype and extracellular matrix remodeling. Clin. Cancer Res..

[57] Ayala G., Tuxhorn J.A., Wheeler T.M., Frolov A., Scardino P.T., Ohori M., Wheeler M., Spitler J., Rowley D.R. (2003). Reactive stroma as a predictor of biochemical-free recurrence in prostate cancer. Clin. Cancer Res..

[58] Olumi A.F., Grossfeld G.D., Hayward S.W., Carroll P.R., Tlsty T.D., Cunha G.R. (1999). Carcinoma-associated fibroblasts direct tumor progression of initiated human prostatic epithelium. Cancer Res..

[59] Pickup M.W., Mouw J.K., Weaver V.M. (2014). The extracellular matrix modulates the hallmarks of cancer. EMBO Rep..

[60] Luthold C., Hallal T., Labbé D.P., Bordeleau F. (2022). The Extracellular Matrix Stiffening: A Trigger of Prostate Cancer Progression and Castration Resistance?. Cancers.

[61] Hawthorne L., Yang J., Zorlutuna P. (2025). Aging and the extracellular matrix: A tumor-permissive microenvironment driving cancer progression. Curr. Opin. Biomed. Eng..

[62] Folkman J. (1971). Tumor angiogenesis: Therapeutic implications. N. Engl. J. Med..

[63] Hanahan D., Folkman J. (1996). Patterns and emerging mechanisms of the angiogenic switch during tumorigenesis. Cell.

[64] Eelen G., de Zeeuw P., Treps L., Harjes U., Wong B.W., Carmeliet P. (2018). Endothelial Cell Metabolism. Physiol. Rev..

[65] Ungvari Z., Tarantini S., Donato A.J., Galvan V., Csiszar A. (2018). Mechanisms of Vascular Aging. Circ. Res..

[66] Potente M., Gerhardt H., Carmeliet P. (2011). Basic and therapeutic aspects of angiogenesis. Cell.

[67] Gilkes D.M., Semenza G.L., Wirtz D. (2014). Hypoxia and the extracellular matrix: Drivers of tumour metastasis. Nat. Rev. Cancer.

[68] Iyengar N.M., Gucalp A., Dannenberg A.J., Hudis C.A. (2016). Obesity and Cancer Mechanisms: Tumor Microenvironment and Inflammation. J. Clin. Oncol..

[69] Ahmad F., Cherukuri M.K., Choyke P.L. (2021). Metabolic reprogramming in prostate cancer. Br. J. Cancer.

[70] Siltari A., Syvälä H., Lou Y.R., Gao Y., Murtola T.J. (2022). Role of Lipids and Lipid Metabolism in Prostate Cancer Progression and the Tumor’s Immune Environment. Cancers.

[71] Scheinberg T., Mak B., Butler L., Selth L., Horvath L.G. (2023). Targeting lipid metabolism in metastatic prostate cancer. Ther. Adv. Med. Oncol..

[72] Pujana-Vaquerizo M., Bozal-Basterra L., Carracedo A. (2024). Metabolic adaptations in prostate cancer. Br. J. Cancer.

[73] Parupathi P., Devarakonda L.S., Francois E., Amjed M., Kumar A. (2025). Reprogrammed Lipid Metabolism-Associated Therapeutic Vulnerabilities in Prostate Cancer. Int. J. Mol. Sci..

[74] Murphy A., Shyanti R.K., Mishra M. (2025). Targeting obesity, metabolic syndrome, and prostate cancer: GLP-1 agonists as emerging therapeutic agents. Discov. Oncol..

[75] Xu X., Pang Y., Fan X. (2025). Mitochondria in oxidative stress, inflammation and aging: From mechanisms to therapeutic advances. Signal Transduct. Target. Ther..

[76] Song Y., Hou Z., Zhu L., Chen Y., Li J. (2025). Oxidative stress as a catalyst in prostate cancer progression: Unraveling molecular mechanisms and exploring therapeutic interventions. Discov. Oncol..

[77] Liu S., Zhang B., Rowan B.G., Jazwinski S.M., Abdel-Mageed A.B., Steele C., Wang A.R., Sartor O., Niu T., Zhang Q. (2021). A Novel Controlled PTEN-Knockout Mouse Model for Prostate Cancer Study. Front. Mol. Biosci..

[78] Liu S., Shen K., Li Z., Rivero C., Zhang Q. (2025). Temporally and Spatially Controlled Age-Related Prostate Cancer Model in Mice. Bio-Protocol.

[79] Zhang Q., Jazwinski S.M. (2022). A Novel Strategy to Model Age-Related Cancer for Elucidation of the Role of Th17 Inflammaging in Cancer Progression. Cancers.

[80] Hirz T., Mei S., Sarkar H., Kfoury Y., Wu S., Verhoeven B.M., Subtelny A.O., Zlatev D.V., Wszolek M.W., Salari K. (2023). Dissecting the immune suppressive human prostate tumor microenvironment via integrated single-cell and spatial transcriptomic analyses. Nat. Commun..

[81] Cheng Y., Liu B., Xin J., Wu X., Li W., Shang J., Wu J., Zhang Z., Xu B., Du M. (2025). Single-cell and spatial RNA sequencing identify divergent microenvironments and progression signatures in early- versus late-onset prostate cancer. Nat. Aging.

[82] Gluck S., Guey B., Gulen M.F., Wolter K., Kang T.W., Schmacke N.A., Bridgeman A., Rehwinkel J., Zender L., Ablasser A. (2017). Innate immune sensing of cytosolic chromatin fragments through cGAS promotes senescence. Nat. Cell Biol..

[83] Dou Z., Ghosh K., Vizioli M.G., Zhu J., Sen P., Wangensteen K.J., Simithy J., Lan Y., Lin Y., Zhou Z. (2017). Cytoplasmic chromatin triggers inflammation in senescence and cancer. Nature.

[84] Xing Y., Qin G., Lin X., Chen S., Su Y., Lin T., Chen G. (2026). The paradox of cellular senescence in prostate cancer: From tumor suppression to tumor promotion. Front. Oncol..

[85] Bacca L., Brandariz J., Zacchi F., Zivi A., Mateo J., Labbe D.P. (2025). Therapy-induced senescence in prostate cancer: Mechanisms, therapeutic strategies, and clinical implications. Gene.

[86] Manda K.R., Tripathi P., Hsi A.C., Ning J., Ruzinova M.B., Liapis H., Bailey M., Zhang H., Maher C.A., Humphrey P.A. (2016). NFATc1 promotes prostate tumorigenesis and overcomes PTEN loss-induced senescence. Oncogene.

[87] Parisotto M., Grelet E., El Bizri R., Dai Y., Terzic J., Eckert D., Gargowitsch L., Bornert J.M., Metzger D. (2018). PTEN deletion in luminal cells of mature prostate induces replication stress and senescence in vivo. J. Exp. Med..

[88] Collado M., Gil J., Efeyan A., Guerra C., Schuhmacher A.J., Barradas M., Benguria A., Zaballos A., Flores J.M., Barbacid M. (2005). Tumour biology: Senescence in premalignant tumours. Nature.

[89] Michaloglou C., Vredeveld L.C., Soengas M.S., Denoyelle C., Kuilman T., van der Horst C.M., Majoor D.M., Shay J.W., Mooi W.J., Peeper D.S. (2005). BRAFE600-associated senescence-like cell cycle arrest of human naevi. Nature.

[90] Baker D.J., Wijshake T., Tchkonia T., LeBrasseur N.K., Childs B.G., van de Sluis B., Kirkland J.L., van Deursen J.M. (2011). Clearance of p16Ink4a-positive senescent cells delays ageing-associated disorders. Nature.

[91] Baker D.J., Childs B.G., Durik M., Wijers M.E., Sieben C.J., Zhong J., Saltness R.A., Jeganathan K.B., Verzosa G.C., Pezeshki A. (2016). Naturally occurring p16(Ink4a)-positive cells shorten healthy lifespan. Nature.

[92] Chen Z., Trotman L.C., Shaffer D., Lin H.K., Dotan Z.A., Niki M., Koutcher J.A., Scher H.I., Ludwig T., Gerald W. (2005). Crucial role of p53-dependent cellular senescence in suppression of Pten-deficient tumorigenesis. Nature.

[93] Alimonti A., Nardella C., Chen Z., Clohessy J.G., Carracedo A., Trotman L.C., Cheng K., Varmeh S., Kozma S.C., Thomas G. (2010). A novel type of cellular senescence that can be enhanced in mouse models and human tumor xenografts to suppress prostate tumorigenesis. J. Clin. Investig..

[94] Di Mitri D., Mirenda M., Vasilevska J., Calcinotto A., Delaleu N., Revandkar A., Gil V., Boysen G., Losa M., Mosole S. (2019). Re-education of Tumor-Associated Macrophages by CXCR2 Blockade Drives Senescence and Tumor Inhibition in Advanced Prostate Cancer. Cell Rep..

[95] Laberge R.M., Sun Y., Orjalo A.V., Patil C.K., Freund A., Zhou L., Curran S.C., Davalos A.R., Wilson-Edell K.A., Liu S. (2015). MTOR regulates the pro-tumorigenic senescence-associated secretory phenotype by promoting IL1A translation. Nat. Cell Biol..

[96] Jin C., Liao S., Lu G., Geng B.D., Ye Z., Xu J., Ge G., Yang D. (2024). Cellular senescence in metastatic prostate cancer: A therapeutic opportunity or challenge. Mol. Med. Rep..

[97] Xu M.Y., Xia Z.Y., Sun J.X., Liu C.Q., An Y., Xu J.Z., Zhang S.H., Zhong X.Y., Zeng N., Ma S.Y. (2024). A new perspective on prostate cancer treatment: The interplay between cellular senescence and treatment resistance. Front. Immunol..

[98] Zhu Y., Tchkonia T., Pirtskhalava T., Gower A.C., Ding H., Giorgadze N., Palmer A.K., Ikeno Y., Hubbard G.B., Lenburg M. (2015). The Achilles’ heel of senescent cells: From transcriptome to senolytic drugs. Aging Cell.

[99] Chang J., Wang Y., Shao L., Laberge R.M., Demaria M., Campisi J., Janakiraman K., Sharpless N.E., Ding S., Feng W. (2016). Clearance of senescent cells by ABT263 rejuvenates aged hematopoietic stem cells in mice. Nat. Med..

[100] Yosef R., Pilpel N., Tokarsky-Amiel R., Biran A., Ovadya Y., Cohen S., Vadai E., Dassa L., Shahar E., Condiotti R. (2016). Directed elimination of senescent cells by inhibition of BCL-W and BCL-XL. Nat. Commun..

[101] Demaria M., O’Leary M.N., Chang J., Shao L., Liu S., Alimirah F., Koenig K., Le C., Mitin N., Deal A.M. (2017). Cellular Senescence Promotes Adverse Effects of Chemotherapy and Cancer Relapse. Cancer Discov..

[102] Xu M., Pirtskhalava T., Farr J.N., Weigand B.M., Palmer A.K., Weivoda M.M., Inman C.L., Ogrodnik M.B., Hachfeld C.M., Fraser D.G. (2018). Senolytics improve physical function and increase lifespan in old age. Nat. Med..

[103] Suda M., Paul K.H., Tripathi U., Minamino T., Tchkonia T., Kirkland J.L. (2024). Targeting Cell Senescence and Senolytics: Novel Interventions for Age-Related Endocrine Dysfunction. Endocr. Rev..

[104] Meguro S., Makabe S., Yaginuma K., Onagi A., Tanji R., Matsuoka K., Hoshi S., Koguchi T., Kayama E., Hata J. (2025). Targeting Senescence in Oncology: An Emerging Therapeutic Avenue for Cancer. Curr. Oncol..

[105] Revandkar A., Perciato M.L., Toso A., Alajati A., Chen J., Gerber H., Dimitrov M., Rinaldi A., Delaleu N., Pasquini E. (2016). Inhibition of Notch pathway arrests PTEN-deficient advanced prostate cancer by triggering p27-driven cellular senescence. Nat. Commun..

[106] Cunha G.R., Hayward S.W., Wang Y.Z., Ricke W.A. (2003). Role of the stromal microenvironment in carcinogenesis of the prostate. Int. J. Cancer.

[107] Kalluri R. (2016). The biology and function of fibroblasts in cancer. Nat. Rev. Cancer.

[108] Barron D.A., Rowley D.R. (2012). The reactive stroma microenvironment and prostate cancer progression. Endocr. Relat. Cancer.

[109] Pacheco-Torres J., Sharma R.K., Mironchik Y., Wildes F., Brennen W.N., Artemov D., Krishnamachary B., Bhujwalla Z.M. (2024). Prostate fibroblasts and prostate cancer associated fibroblasts exhibit different metabolic, matrix degradation and PD-L1 expression responses to hypoxia. Front. Mol. Biosci..

[110] Di Carlo E., Sorrentino C. (2024). The multifaceted role of the stroma in the healthy prostate and prostate cancer. J. Transl. Med..

[111] Thomas R., Jerome J.M., Krieger K.L., Ashraf N., Rowley D.R. (2024). The reactive stroma response regulates the immune landscape in prostate cancer. J. Transl. Genet. Genom..

[112] Chen H., Fang S., Zhu X., Liu H. (2024). Cancer-associated fibroblasts and prostate cancer stem cells: Crosstalk mechanisms and implications for disease progression. Front. Cell Dev. Biol..

[113] Chen P., Chen J., Zhan P., Ye X., Zhao L., Zhang Z., Zuo J., Shi H., Li X., Wu S. (2025). Targeting Cancer-Associated Fibroblasts in Prostate Cancer: Recent Advances and Therapeutic Opportunities. Cancers.

[114] Goronzy J.J., Weyand C.M. (2013). Understanding immunosenescence to improve responses to vaccines. Nat. Immunol..

[115] Pawelec G. (2018). Age and immunity: What is “immunosenescence”?. Exp. Gerontol..

[116] Aiello A., Farzaneh F., Candore G., Caruso C., Davinelli S., Gambino C.M., Ligotti M.E., Zareian N., Accardi G. (2019). Immunosenescence and Its Hallmarks: How to Oppose Aging Strategically? A Review of Potential Options for Therapeutic Intervention. Front. Immunol..

[117] Wang L., Tang D. (2025). Immunosenescence promotes cancer development: From mechanisms to treatment strategies. Cell Commun. Signal.

[118] Zhang Q., Liu S., Ge D., Zhang Q., Xue Y., Xiong Z., Abdel-Mageed A.B., Myers L., Hill S.M., Rowan B.G. (2012). Interleukin-17 promotes formation and growth of prostate adenocarcinoma in mouse models. Cancer Res..

[119] Zhang Q., Liu S., Parajuli K.R., Zhang W., Zhang K., Mo Z., Liu J., Chen Z., Yang S., Wang A.R. (2017). Interleukin-17 promotes prostate cancer via MMP7-induced epithelial-to-mesenchymal transition. Oncogene.

[120] Mei S., Zhang H., Hirz T., Jeffries N.E., Xu Y., Baryawno N., Wu S., Wu C.L., Patnaik A., Saylor P.J. (2025). Single-Cell and Spatial Transcriptomics Reveal a Tumor-Associated Macrophage Subpopulation that Mediates Prostate Cancer Progression and Metastasis. Mol. Cancer Res..

[121] Paszek M.J., Zahir N., Johnson K.R., Lakins J.N., Rozenberg G.I., Gefen A., Reinhart-King C.A., Margulies S.S., Dembo M., Boettiger D. (2005). Tensional homeostasis and the malignant phenotype. Cancer Cell.

[122] Levental K.R., Yu H., Kass L., Lakins J.N., Egeblad M., Erler J.T., Fong S.F., Csiszar K., Giaccia A., Weninger W. (2009). Matrix crosslinking forces tumor progression by enhancing integrin signaling. Cell.

[123] Mai Z., Lin Y., Lin P., Zhao X., Cui L. (2024). Modulating extracellular matrix stiffness: A strategic approach to boost cancer immunotherapy. Cell Death Dis..

[124] Guccini I., Revandkar A., D’Ambrosio M., Colucci M., Pasquini E., Mosole S., Troiani M., Brina D., Sheibani-Tezerji R., Elia A.R. (2021). Senescence Reprogramming by TIMP1 Deficiency Promotes Prostate Cancer Metastasis. Cancer Cell.

[125] Calle E.E., Rodriguez C., Walker-Thurmond K., Thun M.J. (2003). Overweight, obesity, and mortality from cancer in a prospectively studied cohort of U.S. adults. N. Engl. J. Med..

[126] Freedland S.J., Aronson W.J. (2005). Obesity and prostate cancer. Urology.

[127] Allott E.H., Masko E.M., Freedland S.J. (2013). Obesity and prostate cancer: Weighing the evidence. Eur. Urol..

[128] Discacciati A., Orsini N., Wolk A. (2012). Body mass index and incidence of localized and advanced prostate cancer--a dose-response meta-analysis of prospective studies. Ann. Oncol..

[129] Freedland S.J., Grubb K.A., Yiu S.K., Humphreys E.B., Nielsen M.E., Mangold L.A., Isaacs W.B., Partin A.W. (2005). Obesity and risk of biochemical progression following radical prostatectomy at a tertiary care referral center. J. Urol..

[130] Qu Y., Zhang Q., Ma S., Liu S., Chen Z., Mo Z., You Z. (2016). Interleukin-17A Differentially Induces Inflammatory and Metabolic Gene Expression in the Adipose Tissues of Lean and Obese Mice. Int. J. Mol. Sci..

[131] Liu S., Zhang Q., Chen C., Ge D., Qu Y., Chen R., Fan Y.M., Li N., Tang W.W., Zhang W. (2016). Hyperinsulinemia enhances interleukin-17-induced inflammation to promote prostate cancer development in obese mice through inhibiting glycogen synthase kinase 3-mediated phosphorylation and degradation of interleukin-17 receptor. Oncotarget.

[132] Swinnen J.V., Esquenet M., Goossens K., Heyns W., Verhoeven G. (1997). Androgens stimulate fatty acid synthase in the human prostate cancer cell line LNCaP. Cancer Res..

[133] Ettinger S.L., Sobel R., Whitmore T.G., Akbari M., Bradley D.R., Gleave M.E., Nelson C.C. (2004). Dysregulation of sterol response element-binding proteins and downstream effectors in prostate cancer during progression to androgen independence. Cancer Res..

[134] Zadra G., Ribeiro C.F., Chetta P., Ho Y., Cacciatore S., Gao X., Syamala S., Bango C., Photopoulos C., Huang Y. (2019). Inhibition of de novo lipogenesis targets androgen receptor signaling in castration-resistant prostate cancer. Proc. Natl. Acad. Sci. USA.

[135] Zadra G., Photopoulos C., Loda M. (2013). The fat side of prostate cancer. Biochim. Biophys. Acta.

[136] Kennedy B.K., Berger S.L., Brunet A., Campisi J., Cuervo A.M., Epel E.S., Franceschi C., Lithgow G.J., Morimoto R.I., Pessin J.E. (2014). Geroscience: Linking aging to chronic disease. Cell.

[137] Taylor B.S., Schultz N., Hieronymus H., Gopalan A., Xiao Y., Carver B.S., Arora V.K., Kaushik P., Cerami E., Reva B. (2010). Integrative genomic profiling of human prostate cancer. Cancer Cell.

[138] Network C.G.A.R. (2015). The Molecular Taxonomy of Primary Prostate Cancer. Cell.

[139] Robinson D., Van Allen E.M., Wu Y.M., Schultz N., Lonigro R.J., Mosquera J.M., Montgomery B., Taplin M.E., Pritchard C.C., Attard G. (2015). Integrative Clinical Genomics of Advanced Prostate Cancer. Cell.

[140] Armenia J., Wankowicz S.A.M., Liu D., Gao J., Kundra R., Reznik E., Chatila W.K., Chakravarty D., Han G.C., Coleman I. (2018). The long tail of oncogenic drivers in prostate cancer. Nat. Genet..

[141] Abida W., Cyrta J., Heller G., Prandi D., Armenia J., Coleman I., Cieslik M., Benelli M., Robinson D., Van Allen E.M. (2019). Genomic correlates of clinical outcome in advanced prostate cancer. Proc. Natl. Acad. Sci. USA.

[142] Tan S.H., Petrovics G., Srivastava S. (2018). Prostate Cancer Genomics: Recent Advances and the Prevailing Underrepresentation from Racial and Ethnic Minorities. Int. J. Mol. Sci..

[143] Kantoff Philip W., Higano Celestia S., Shore Neal D., Berger E.R., Small Eric J., Penson David F., Redfern Charles H., Ferrari Anna C., Dreicer R., Sims Robert B. (2010). Sipuleucel-T Immunotherapy for Castration-Resistant Prostate Cancer. N. Engl. J. Med..

[144] Kwon E.D., Drake C.G., Scher H.I., Fizazi K., Bossi A., van den Eertwegh A.J., Krainer M., Houede N., Santos R., Mahammedi H. (2014). Ipilimumab versus placebo after radiotherapy in patients with metastatic castration-resistant prostate cancer that had progressed after docetaxel chemotherapy (CA184-043): A multicentre, randomised, double-blind, phase 3 trial. Lancet Oncol..

[145] Beer T.M., Kwon E.D., Drake C.G., Fizazi K., Logothetis C., Gravis G., Ganju V., Polikoff J., Saad F., Humanski P. (2017). Randomized, Double-Blind, Phase III Trial of Ipilimumab Versus Placebo in Asymptomatic or Minimally Symptomatic Patients with Metastatic Chemotherapy-Naive Castration-Resistant Prostate Cancer. J. Clin. Oncol..

[146] Antonarakis E.S., Piulats J.M., Gross-Goupil M., Goh J., Ojamaa K., Hoimes C.J., Vaishampayan U., Berger R., Sezer A., Alanko T. (2020). Pembrolizumab for Treatment-Refractory Metastatic Castration-Resistant Prostate Cancer: Multicohort, Open-Label Phase II KEYNOTE-199 Study. J. Clin. Oncol..

[147] Sharma P., Pachynski R.K., Narayan V., Flechon A., Gravis G., Galsky M.D., Mahammedi H., Patnaik A., Subudhi S.K., Ciprotti M. (2020). Nivolumab Plus Ipilimumab for Metastatic Castration-Resistant Prostate Cancer: Preliminary Analysis of Patients in the CheckMate 650 Trial. Cancer Cell.

[148] Sun Y., Campisi J., Higano C., Beer T.M., Porter P., Coleman I., True L., Nelson P.S. (2012). Treatment-induced damage to the tumor microenvironment promotes prostate cancer therapy resistance through WNT16B. Nat. Med..

[149] Fleshner N.E., Bernardino R.M., Izawa J., Drachenberg D., Saranchuk J.W., Fairey A., Tanguay S., Leveridge M., Saad F., Breau R.H. (2025). Metformin Active Surveillance Trial in Low-Risk Prostate Cancer. J. Clin. Oncol..

[150] Chandran K., Guo C., Pacey S., Villacampa G., Matthews R., Prout T., Fernandes J., Turner A., Parmar M., Riisnaes R. (2026). A Phase I trial of the anti-IL23 monoclonal antibody tildrakizumab, in combination with abiraterone acetate, for the treatment of metastatic castration-resistant prostate cancer. Investig. New Drugs.

[151] National Cancer Institute Ruxolitinib and Enzalutamide for the Treatment of Metastatic Castration-Resistant Prostate Cancer. https://www.cancer.gov/research/participate/clinical-trials-search/v?id=NCI-2024-06533.

[152] Tuong Z.K., Loudon K.W., Berry B., Richoz N., Jones J., Tan X., Nguyen Q., George A., Hori S., Field S. (2021). Resolving the immune landscape of human prostate at a single-cell level in health and cancer. Cell Rep..

[153] Wong H.Y., Sheng Q., Hesterberg A.B., Croessmann S., Rios B.L., Giri K., Jackson J., Miranda A.X., Watkins E., Schaffer K.R. (2022). Single cell analysis of cribriform prostate cancer reveals cell intrinsic and tumor microenvironmental pathways of aggressive disease. Nat. Commun..

[154] National Cancer Institute Genetics of Prostate Cancer (PDQ)-Health Professional Version. https://www.cancer.gov/types/prostate/hp/prostate-genetics-pdq.

[155] Pernigoni N., Guo C., Gallagher L., Yuan W., Colucci M., Troiani M., Liu L., Maraccani L., Guccini I., Migliorini D. (2023). The potential role of the microbiota in prostate cancer pathogenesis and treatment. Nat. Rev. Urol..

[156] Salachan P.V., Rasmussen M., Fredsøe J., Ulhøi B., Borre M., Sørensen K.D. (2022). Microbiota of the prostate tumor environment investigated by whole-transcriptome profiling. Genome Med..

[157] Olivieri F., Prattichizzo F., Giuliani A., Matacchione G., Rippo M.R., Sabbatinelli J., Bonafè M. (2021). miR-21 and miR-146a: The microRNAs of inflammaging and age-related diseases. Ageing Res. Rev..

[158] Olivieri F., Rippo M.R., Monsurrò V., Salvioli S., Capri M., Procopio A.D., Franceschi C. (2013). MicroRNAs linking inflamm-aging, cellular senescence and cancer. Ageing Res. Rev..

[159] Hickson L.J., Langhi Prata L.G.P., Bobart S.A., Evans T.K., Giorgadze N., Hashmi S.K., Herrmann S.M., Jensen M.D., Jia Q., Jordan K.L. (2019). Senolytics decrease senescent cells in humans: Preliminary report from a clinical trial of Dasatinib plus Quercetin in individuals with diabetic kidney disease. EBioMedicine.

[160] Justice J.N., Nambiar A.M., Tchkonia T., LeBrasseur N.K., Pascual R., Hashmi S.K., Prata L., Masternak M.M., Kritchevsky S.B., Musi N. (2019). Senolytics in idiopathic pulmonary fibrosis: Results from a first-in-human, open-label, pilot study. eBioMedicine.

